# The neuropsychopharmacology of cannabis: A review of human imaging studies

**DOI:** 10.1016/j.pharmthera.2018.10.006

**Published:** 2019-03

**Authors:** Michael A.P. Bloomfield, Chandni Hindocha, Sebastian F. Green, Matthew B. Wall, Rachel Lees, Katherine Petrilli, Harry Costello, M. Olabisi Ogunbiyi, Matthijs G. Bossong, Tom P. Freeman

**Affiliations:** a Translational Psychiatry Research Group, Research Department of Mental Health Neuroscience, Division of Psychiatry, Faculty of Brain Sciences, University College London, United Kingdom; b Clinical Psychopharmacology Unit, Research Department of Clinical, Educational and Health Psychology, Faculty of Brain Sciences, University College London, United Kingdom; c Psychiatric Imaging Group, MRC London Institute of Medical Sciences, Hammersmith Hospital, London, United Kingdom; d NIHR University College London Hospitals Biomedical Research Centre, University College Hospital, London, United Kingdom; e Institute of Clinical Sciences, Faculty of Medicine, Imperial College London, United Kingdom; f Centre for Neuropsychopharmacology, Division of Brain Sciences, Faculty of Medicine, Imperial College London, United Kingdom; g Invicro UK, Hammersmith Hospital, London, United Kingdom; h Institute of Cognitive Neuroscience, Faculty of Brain Sciences, University College London, United Kingdom; i Department of Psychiatry, Brain Center Rudolf Magnus, University Medical Center Utrecht, the Netherlands; j Department of Psychology, University of Bath, United Kingdom; k National Addiction Centre, Institute of Psychiatry, Psychology & Neuroscience, King’s College London, United Kingdom

**Keywords:** Addiction, Cannabis, Cognition, Development, Neuroimaging, Psychosis, ACC, Anterior cingulate cortex, ASL, Arterial spin labelling, BOLD, Blood-oxygen-level dependent, CBD, Cannabidiol, CBF, Cerebral blood flow, CB_1_R, Endocannabinoid type 1 receptor, CT, Computed tomography, D_2_R, Dopamine type 2 receptor, DLPFC, Dorsolateral prefrontal cortex, DTI, Diffusion tensor imaging, EEG, Electroencephalography, OFC, Orbitofrontal cortex, FDG, Fludeoxyglucose, fMRI, Functional magnetic resonance imaging, GABA, γ-Aminobutyric acid, MID, Monetary incentive delay, MRI, Magnetic resonance imaging, MRS, Magnetic resonance spectroscopy, NAA, N-Acetylaspartate, NAc, Nucleus accumbens, PCC, Posterior cingulate cortex, PET, Positron emission tomography, PFC, Prefrontal cortex, THC, Δ^9^-Tetrahydrocannabinol

## Abstract

The laws governing cannabis are evolving worldwide and associated with changing patterns of use. The main psychoactive drug in cannabis is Δ^9^-tetrahydrocannabinol (THC), a partial agonist at the endocannabinoid CB_1_ receptor. Acutely, cannabis and THC produce a range of effects on several neurocognitive and pharmacological systems. These include effects on executive, emotional, reward and memory processing via direct interactions with the endocannabinoid system and indirect effects on the glutamatergic, GABAergic and dopaminergic systems. Cannabidiol, a non-intoxicating cannabinoid found in some forms of cannabis, may offset some of these acute effects. Heavy repeated cannabis use, particularly during adolescence, has been associated with adverse effects on these systems, which increase the risk of mental illnesses including addiction and psychosis. Here, we provide a comprehensive state of the art review on the acute and chronic neuropsychopharmacology of cannabis by synthesizing the available neuroimaging research in humans. We describe the effects of drug exposure during development, implications for understanding psychosis and cannabis use disorder, and methodological considerations. Greater understanding of the precise mechanisms underlying the effects of cannabis may also give rise to new treatment targets.

## 1 Introduction

Cannabis is one of the most widely used recreational drugs in the world (United Nations Office on Drugs and Crime (UNODC), 2018). The past year prevalence of cannabis use disorders in the United States has been estimated at 2.9%, or 30.6% among past-year users (Hasin et al., 2015). There has been concern over the link between cannabis use and psychiatric illness since the 1960s (Advisory Committee on Drug Dependence, 1969; Kolansky & Moore, 1972; Tennant & Groesbeck, 1972), which has intensified following a series of large scale epidemiological studies (Andreasson et al. 1987; Murray et al., 2007) and wide public debate. A changing legal landscape for the drug has been associated with increasing usage and reductions in the perception of harm (Cerdá et al., 2017). Acute intoxication and chronic heavy use of cannabis have been associated with a range of effects. The potential long-term deleterious effects of particular concern are when heavy cannabis use occurs during adolescence, a key developmental period for the brain (Bossong & Niesink, 2010). Positive subjective acute effects described as the ‘high’ include euphoria, relaxation and sensory intensification (Green et al., 2003). Adverse acute effects include anxiety, paranoia, impaired psychomotor performance and cognitive dysfunction (Broyd et al., 2016; Curran et al., 2016). Chronic heavy use of the drug is associated with increased risk of dependence, psychosis and cognitive impairment (Broyd et al., 2016; Curran et al., 2016; Marconi et al., 2016). However, two large meta-analyses suggest that the adverse effects of chronic cannabis use on cognition may improve following abstinence (Schreiner & Dunn, 2012; Scott et al., 2018).

**Fig. 1:**
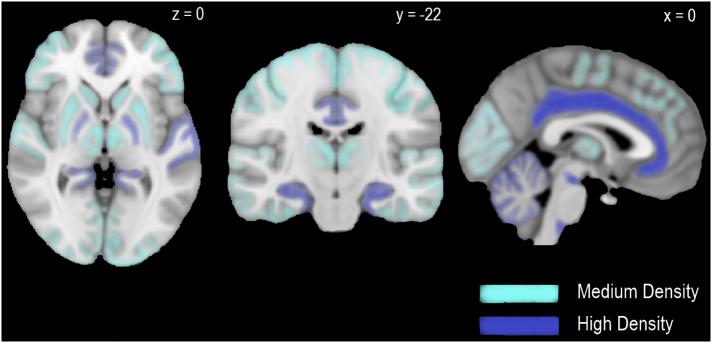
The distribution of CB_1_Rs across the human brain. These axial (left), coronal (middle) and sagittal (right) views schematically depict regions of medium and high endocannabinoid type 1 receptor (CB_1_R) concentration. This was extrapolated from mean labelling densities as described by Glass et al. (1997). [^3^H]CPP55,940 binding >80 fmol/mg was defined as high and 40-80 fmol/mg was defined as medium. Regions with high CB_1_R concentration include (in alphabetical order): amygdala (not in view), cerebellum, cingulate gyrus, dorsal motor nucleus of the vagus, entorhinal cortex, globus pallidus, hippocampal formation, middle frontal gyrus, substantia nigra, and Wernicke’s area. Regions with medium CB_1_R concentration include (in alphabetical order): auditory cortex (right), caudate nucleus, mediodorsal nucleus of the thalamus, motor cortex, occipitotemporal gyrus, putamen, somatosensory cortex, and visual cortex. Montreal Neurological Institute coordinates (x,y,z) are shown above.

**Fig. 2:**
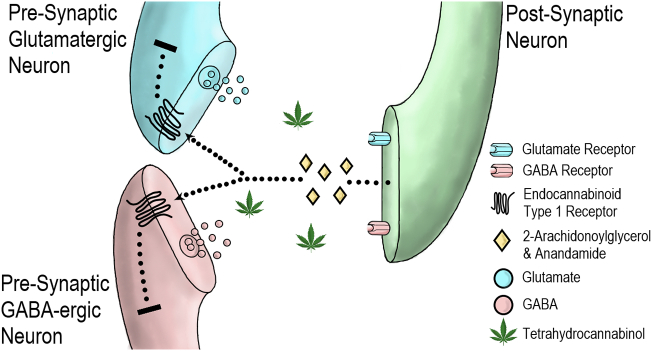
THC and retrograde endocannabinoid signalling at the synaptic cleft. The cannabinoids 2-arachidonoylglycerol and anandamide are produced endogenously by neurons and act at endocannabinoid type 1 receptors (CB_1_Rs) on adjacent synaptic terminals. CB_1_R activity leads to retrograde suppression of excitation in glutamatergic nerve terminals and retrograde suppression of inhibition in GABAergic nerve terminals. Δ^9^-tetrahydrocannabinol (THC) disrupts this signalling process.

The main psychoactive substance in cannabis is Δ^9^-tetrahydrocannabinol (THC) (Wachtel et al., 2002) which was first isolated from hashish in 1964 by Gaoni and Mechloulam. THC is gaining interest for its broad therapeutic potential. This includes putative anti-epileptic properties (Friedman & Devinsky, 2015), analgesic properties in neuropathic and chronic pain (Abrams et al., 2007; Mucke et al., 2018; Narang et al., 2008; Svendsen et al., 2004; Wilsey et al., 2008), anti-emetic properties in cancer (Davis, 2016; Smith, Azariah, et al., 2015), and anti-spastic properties in stroke and multiple sclerosis (Collin et al., 2007; Marinelli et al., 2017). THC was originally described as an agonist of endocannabinoid CB_1_ receptors (CB_1_R) (Felder et al. 1992), however, there is growing evidence of partial agonist properties at this site from both in vitro (Breivogel & Childers, 2000; Govaerts et al., 2004; Kelley & Thayer, 2004; Petitet et al., 1998; Shen & Thayer, 1999; Sim et al., 1996) and in vivo (Paronis et al., 2012) studies. The CB_1_R is a widespread G protein-coupled receptor (Pertwee, 2008) found at high concentrations in key brain regions associated with reward, emotional and cognitive processing including the neocortex (particularly frontal and limbic areas), hippocampus, amygdala, cerebellum, thalamus and basal ganglia (see Fig. 1) (Glass et al., 1997). THC alters signalling of endocannabinoid transmitters such as 2-arachidonoylglycerol and anandamide. These ligands are released endogenously by neurons and act on CB_1_Rs in adjacent γ-aminobutyric acid (GABA)-ergic and glutamatergic nerve terminals resulting in retrograde signalling (see Fig. 2) (Bloomfield et al., 2016; Castillo et al., 2012). THC also demonstrates partial agonist properties in vitro at the CB_2_ receptor, but with lower efficacy than at CB_1_R. (Pertwee, 2008). As THC has a number of double bonds and stereoisomers, this review focuses on the main THC isomer found in cannabis, (−)-trans-Δ^9^-tetrahydrocannabinol, which is also referred to in some older studies by its alternative name Δ^1^-tetrahydrocannabinol and as a pharmaceutical preparation using the International Non-Proprietary Name dronabinol.

The cannabis plant synthesises at least 143 other cannabinoids in addition to THC (Hanuš et al. 2016) such as cannabidiol (CBD). With its excellent safety and tolerability profile and lack of intoxicating effects, CBD has generated significant interest as a novel treatment for psychosis, (Leweke et al., 2012; McGuire et al., 2017) epilepsy (Devinsky et al., 2017; Devinsky et al., 2018), anxiety disorders (Bergamaschi et al., 2011; Crippa et al., 2004) and addictions (Hindocha, Freeman, et al., 2018; Morgan et al., 2013; Ren et al., 2009). When administered alone, CBD has minimal activity at CB_1_Rs, but it can inhibit the effects of cannabinoid agonists by acting as a negative allosteric modulator of CB_1_Rs (Laprairie et al., 2015). Moreover, CBD can inhibit the reuptake and hydrolysis of the endocannabinoid anandamide (Bisogno et al., 2001). CBD has many additional targets within and beyond the endocannabinoid system, including activation of 5-HT_1A_ receptors, α_1_-adrenoceptors and μ-opioid receptors (for a review see Pertwee, 2008). Whilst a balance of THC and CBD is typically found in hashish or resin products produced by landrace crops, cannabis plants are increasingly selected to produce THC only (Potter et al. 2008). The acute harms of THC are dose-dependent (Curran et al., 2002; D'Souza et al., 2004) and may be offset by CBD (Bhattacharyya et al., 2010; Englund et al., 2013; Hindocha et al., 2015; Morgan et al., 2010). THC levels and the THC:CBD ratio in cannabis have risen considerably in the USA and Europe in the last two decades (ElSohly et al., 2016; Pijlman et al., 2005; Potter et al., 2018; Zamengo et al., 2015), which may increase the harms from repeated use (Di Forti et al., 2015; Freeman & Winstock, 2015; Freeman, van der Pol, et al., 2018; Schoeler et al., 2016). In this article, we refer to cannabis containing THC only or with unknown quantities of CBD as ‘cannabis’, and we explicitly state when cannabis contains significant levels of CBD.

Cannabis and THC can induce transient positive psychotic symptoms in healthy individuals (Bhattacharyya et al., 2010; D'Souza et al., 2004; Moreau, 1845; Morrison & Stone, 2011; Morrison et al., 2009; Morrison et al., 2011). Increased sensitivity to the acute psychotogenic effects of cannabis has been found in people with higher schizotypal personality traits (Mason et al., 2009) and those with genetic vulnerability (Morgan et al. 2016). This increased sensitivity also has been shown to be a predictor of subsequent psychotic disorders (Arendt et al., 2005). THC can also elicit schizophreniform negative symptoms which are distinct from sedation (Morrison & Stone, 2011). There is consistent epidemiological evidence that the drug is a risk factor for schizophreniform psychotic disorders (Di Forti et al., 2015), exhibiting dose-dependence (Gage et al., 2016; Marconi et al., 2016; Moore et al., 2007) and dose-duration effects (Di Forti et al., 2009). Even in cannabis users who do not have frank schizophrenia, drug use is associated with increased paranoia; (Freeman et al., 2015; Freeman et al., 2013) a cardinal symptom of the illness. The available evidence indicates that cannabis causes psychosis in susceptible individuals (Murray et al., 2007). However, there is some evidence to suggest that causal effects of cannabis on risk of psychosis may be smaller than reverse causation from psychosis risk to cannabis use (Gage et al., 2016; Pasman et al., 2018).

Studies in non-human animals show that THC produces morphological changes in brain regions with high CB_1_R expression including the hippocampus (Chan et al., 1998), amygdala (Heath et al. 1980) and cortex (Downer et al. 2001). These include reductions in synapses (Heath et al., 1980), cell body size (Scallet et al., 1987) and dendritic length (Landfield et al., 1988). Additionally, THC and cannabis produce complex effects on neuropharmacology including the dopaminergic system (Bloomfield et al., 2016). Alterations in brain structure and function have also been found in human cannabis users, particularly in CB_1_R-rich areas of the brain that support executive, memory and emotional processing (Lorenzetti, Solowij, and Yucel, 2016; Yücel et al., 2007).

Heavy cannabis use has been associated with a range of neurocognitive effects of relevance to mental illness, which may persist after acute intoxication (Broyd et al., 2016; Curran et al., 2016; Volkow et al., 2016). These include negative effects on attention (Crane et al., 2013), executive function (Crean et al., 2011), learning (Crane et al., 2013), memory (Jager et al., 2010), psychotic experiences (D'Souza et al., 2004; Fletcher & Honey, 2006), anhedonia and anxiety (Dorard et al., 2008). These deficits may be reversible as a meta-analysis of neurocognitive performance after at least 25 days of abstinence from cannabis found no evidence of impairment (Schreiner & Dunn, 2012). An additional meta-analysis of 69 studies found that cognitive impairments in frequent users were of a small effect size, and found no evidence for impairment after more than 72 hours of abstinence (Scott et al., 2018).

It is thus timely to review the human imaging literature on the neuropsychopharmacology of cannabis. We build upon and extend recent review articles (Blest-Hopley et al., 2018; Lorenzetti, Alonso-Lana, et al., 2016; Weinstein et al., 2016; Yanes et al., 2018) by incorporating multiple structural, functional, and pharmacological neuroimaging modalities with a focus on both the adolescent and adult brain to present a comprehensive overview of the neuropsychopharmacology of cannabis. We will begin by describing the effects of acute pharmacological challenge of either cannabis or THC before considering neuroimaging studies of heavy cannabis users. As our focus is on cannabis we will omit imaging studies of synthetic cannabinoids (sometimes referred to collectively as “spice”). We will give additional consideration to the neuropharmacology of cannabis during development because CB_1_R expression peaks during the foetal period and adolescence (Jacobus et al., 2014), key periods associated with neuroanatomical re-modelling (Bossong & Niesink, 2010; Raznahan et al., 2014). This is because of potential harms associated with maternal cannabis exposure during gestation and breast-feeding, and because adolescence and young adulthood is the period of peak cannabis use (Copeland et al., 2013), and may be a particularly vulnerable period to the acute effects of cannabinoids (Curran et al., 2016). Given the public health implications, we will synthesise the literature on implications for understanding psychosis and cannabis use disorder before describing important methodological considerations.

## 2 Methodology

For this narrative review, a series of searches of the electronic databases PubMed, Medline, and Ovid were conducted to identify relevant studies between 1966 and (19^th^ September) 2018. Google Scholar updates were used for search terms ‘cannabis’, ‘marijuana’, ‘THC’, and key papers were manually searched to identify further studies. The following search terms were used: ‘cannabis’; ‘THC’; ‘Δ^9^-tetrahydrocannabinol’; ‘Δ^1^-tetrahydrocannabinol’; ‘dronabinol’; ‘tetrahydrocannabinol’; ‘marijuana’; ‘endocannabinoid’; ‘cannabinoid’; ‘CB1’; ‘glutamate’; ‘glutamatergic’; ‘GABA’; ‘gamma-aminobutyric acid’; ‘dopamine’; ‘dopaminergic’; ‘N-acetylaspartate’; ‘neuropsychopharmacology’; ‘pharmacology’; ‘functional magnetic resonance imaging’; ‘fMRI’; ‘blood oxygen level dependent’; ‘BOLD’; ‘diffusion tensor tractography’; ‘DTT’; ‘diffusion tensor imaging’; ‘DTI’; ‘spectroscopy’; ‘electroencephalography’; ‘EEG’; ‘computed tomography’; ‘CT’; ‘single photon emission tomography’; ‘SPECT’; ‘positron emission tomography’; ‘PET’; ‘neuroimaging’; ‘brain imaging’; ‘brain structure’; ‘cerebral blood flow’; ‘cerebral perfusion’; ‘brain volume’; ‘attention’; ‘salience’; ‘awareness’; ‘response inhibition’; ‘reward’; ‘executive function’; ‘learning’; ‘memory’; ‘recall’; ‘amnesia’; ‘emotion’; ‘affect’; ‘decision’; ‘cognition’; ‘cognitive impairment’; ‘brain activity’; ‘psychomotor’; ‘movement’; “brain function; ‘psychosis’; ‘schizophrenia’; ‘psychotomimetic’; ‘adolescent’; ‘young adult’; ‘brain maturation’; ‘brain development’; ‘neurodevelopment’. There was no language restriction. Articles were only included if they were directly related to the topic and employed a quantitative research design.

## 3 The acute effects of cannabis and THC

### 3.1 Cerebral blood flow and metabolism

The first neuroimaging studies using acute cannabinoid challenge were a series of experiments using ^133^Xe inhalation cerebral blood flow tomography. Acutely, THC alters global and regional cerebral blood flow (CBF) (Mathew et al., 1989; Mathew et al. 1992a; Mathew et al. 1992b; Mathew & Wilson, 1993). Nearly every study using H_2_[^15^O]- positron emission tomography (PET) found THC-induced increases in CBF in the frontal cortex, insula and cingulate gyrus (Mathew et al., 1997; Mathew et al., 1998; Mathew et al., 1999; Mathew et al., 2002; O'leary et al., 2000; O'Leary et al., 2002; O'leary et al., 2007). In contrast, one hour after smoking a ‘joint’, decreases in cortical CBF were observed. Importantly, these pioneering studies found relationships between cannabinoid-induced increases in CBF and subjective intoxication, dissociation, depersonalisation and confusion (Mathew et al., 1992b; Mathew et al., 1993). Subsequently, magnetic resonance imaging (MRI) measures of CBF such as arterial spin labelling (ASL) have corroborated the PET findings (van Hell et al., 2011). In terms of metabolism, using [^18^F]-deoxyglucose (FDG) PET, Volkow et al. (1996) demonstrated that acute THC increased metabolism in the basal ganglia and the orbitofrontal cortex (OFC) and prefrontal cortex (PFC). Taken together, these studies indicate that acute THC causes region-specific increases in CBF and metabolism, particularly in frontal regions (Table 1).

**Table 1:** Neuroimaging studies of the acute effects of THC and cannabis on cerebral blood flow and metabolism, and resting state networks.

Author	Imaging Modality	User Groups	Group Sample Size (n)	Group Definition	Drug	Task	User Age Mean (SD)	Dose of THC	Route	Increase (volume, blood flow, activation, connectivity)	Decrease (volume, blood flow, activation, connectivity)	Task Performance (THC vs comparison group or baseline)
Acute effects on cerebral blood flow and metabolism												
Mathew et al. (1989)	^133^Xe SPECT	O/Fr/C	17/9/14	O = no cannabis for 3y; Fr = ≥10 joints/week for 3y; C = unknown cannabis history	Cannabis	Resting	28.3 (8.3)	2.20%	S	Frontal, L temporal (chronic users only)	Baseline global CBF (chronic users only)	-
Mathew et al. (1992a)	^133^Xe SPECT	O	20	O = unclear previous use	Cannabis	Resting	25.3 (6.4)	1.75% or 3.55%	S	R Frontal, R temporal	-	-
Mathew et al. (1992a)	TCD	O	10	O = unclear previous use	Cannabis	Resting	25.9 (6)	3.55%	S	Middle Cerebral Artery	-	-
Mathew & Wilson (1993)	^133^Xe SPECT	Fr	35	Fr = unclear previous use	Cannabis	Resting	21.7 (8)	1.75% or 3.55%	S	Global CBF, R Frontal	-	-
Volkow et al. (1996)	^18^F-FDG PET	O/Fr	8/8	Fr = DSM-III criteria for cannabis dependence, used for >18m, used for mean 5.5y, r1-7d/w; O = used cannabis <twice/y	THC	Resting	31 (6)	2mg	IV	Basal banglia, OFC, PFC	Cerebellum (chronic users)	-
Mathew et al. (1997)	H_2_^15^0 PET	O	32	O = mean onset age 15.7 (M) 17.6 (F)	THC	Resting	32.5 (7.6)	3mg or 5mg	IV	Global CBF, frontal cortex, R insula, R cingulate gyrus, R subcortical regions	Frontal CBF at 1 hour.	-
Mathew et al. (1998)	H_2_^15^0 PET	Fr	46	O = mean 147 (SD 165.2) joints/y	THC	Resting	29.9 (6.5)	3mg or 5mg	IV	ACC, insula, cerebellum	Cerebellum	-
Mathew et al. (1999)	H_2_^15^0 PET	O	59	O = mean onset age 16.8 (3.6)y	THC	Resting	31.8 (7.5)	3mg or 5mg	IV	Global CBF (R>L), R frontal, R insula, ACC	Basal ganglia, thalamus, HPC, amygdala	-
O'leary et al. (2000)	H_2_^15^0 PET	O	5	O = use <10 times/m for mean 3.2y	Cannabis	Auditory Attention Task	26.2 (8)	20mg	S	OFC, insula, temporal poles, ACC, cerebellum	Auditory cortex	No significant change
Mathew et al. (2002)	H_2_^15^0 PET	Fr	47	Fr = mean 228.3 (SD 416.8) joints/y, no dependence by DSM-III criteria	THC	Resting	32.0 (8.3)	3mg or 5mg	IV	Global CBF (R>L, A>P), R insular, R ACC, cerebellum (5mg only)	-	-
O'Leary et al. (2002)	H_2_^15^0 PET	O	12	O = use <10 times/m, mean 2.7 times/m	Cannabis	Auditory Attention Task	30.5 (8.6)	20mg	S	MPFC, insula, temporal poles, ACC, cerebellum	Auditory cortex, Visual cortex, Attentional Network (parietal, frontal, thalamus)	No significant change
O'leary et al. (2007)	H_2_^15^0 PET	O	12	O = use <10 times/m, mean 5.1 times/m, duration mean 3.1y	Cannabis	Auditory Attention Task	23.5 (4.3)	20mg	S	OFC, ACC, temporal pole, insula, cerebellum	Auditory cortex, Visual cortex	No significant change
van Hell et al. (2011)	ASL & fMRI	O	26	O = mean use 19.0 (SD 11.2) in last year	THC	Resting	21.1 (2.1)	6mg	INH	ACC, superior frontal cortex, insula, substantia nigra, cerebellum	Post-central gyrus, occipital gyrus	-

Acute effects on resting state networks												
Klumpers et al. (2012)	fMRI	O	12	O = >1y of use duration, ≤1 use/w	THC	Resting	22 (2.9)	2mg or 6mg	INH	sensorimotor network, dorsal-visual streams	R superior frontal pole - middle and inferior frontal gyri - PFC network	-
Ramaekers et al. (2016)	fMRI	Fr	122	Fr = mean use 7y duration, mean 44.8 uses in last 3m	THC	Resting	22.8 (3.7)	450μg/kg	INH	-	NAc - PFC, limbic lobe, striatum, thalamus	-
Grimm et al. (2018)	fMRI	O	16	≤5 uses in lifetime	THC	Resting	Range 18-50	10mg	PO	No significant changes		-

ACC = anterior cingulate cortex, ASL = arterial spin labelling, C = control users, CBF = cerebral blood flow, d = day, DSM = Diagnostic & Statistic Manual of Mental Disorders, Fr = frequent cannabis users, F = female, fMRI = functional magnetic resonance imaging, HPC = hippocampus, INH = inhaled, IV = intravenous, L = left, m = month, M = male, MPFC = medial prefrontal cortex, NAc = nucleus accumbens, O = occasional cannabis users, OFC = orbitofrontal cortex, PFC = prefrontal cortex, PO = per os (oral), PET = positron emission tomography, r = range, R = right, S = smoked, SD = standard deviation, SPECT = single photon emission computed tomography, TCD = transcranial doppler, THC = Δ^9^-tetrahydrocannabinol, VMPFC = ventromedial prefrontal cortex, w = week, y = year.

### 3.2 Resting state networks

In healthy volunteers, THC inhalation (2 mg or 6 mg) vs. placebo, increased functional connectivity in the sensorimotor network and dorsal visual streams alongside reduced connectivity in the right hemisphere between the superior frontal pole, middle and inferior frontal gyri and dorsolateral prefrontal cortex (DLPFC) (Klumpers et al., 2012). However, that study was compromised by a 41% drop-out rate during THC challenge, particularly in women. Post-hoc analysis suggested this may have been due to higher peak plasma THC concentrations in women compared to men. Another study found no effects of 10 mg oral THC on frontostriatal connectivity in healthy volunteers (Grimm, et al., 2018). However, this may have been attributable to low concentrations of THC during scanning. In the same study, the authors found that CBD (600 mg oral) increased frontostriatal connectivity. THC-induced changes in functional connectivity have also been observed in regular drug users, whereby THC (450 micrograms/kg inhaled) resulted in reduced functional connectivity between the nucleus accumbens (NAc) and the PFC, limbic lobe, striatum and thalamus in a manner similar to acute cocaine (300 mg oral; Ramaekers et al., 2016). Importantly, those results were moderated by dopamine beta-hydroxylase enzyme genotype, with CC/TT (low activity) carriers showing greatest reduction in functional connectivity. Moreover, sub-cortical functional connectivity was inversely related to impulsivity scores on the matching familiar figures test, indicating that those who experienced greater reductions in functional connectivity following THC showed increased impulsivity at the behavioural level (Table 1).

### 3.3 Attentional processing

Acute cannabis inhalation reduces CBF during the performance of focused attention tasks (dichotic listening and auditory reaction time tasks) in visual and auditory cortices (O'Leary et al., 2002; O'leary et al., 2007), and brain regions that are part of the attentional network (parietal lobe, frontal lobe, and thalamus) (O'Leary et al., 2002). Using a visual oddball task, 10mg oral THC increased activation in the right PFC, attenuated activation in the right caudate and increased response latency to oddball stimuli (Bhattacharyya et al., 2012). There was a negative relationship between THC-induced caudate hypoactivation and both psychotic symptoms and effects on response latency. That study also included a CBD challenge which found opposite effects compared to THC alongside hippocampal hyper-activation. Acute inhaled vaporised THC (6mg), compared to placebo, resulted in increased false alarms and reduced target detection during a continuous performance of sustained attention task (Bossong, Jansma, et al., 2013). Impaired task performance was related to impaired deactivation of default mode regions including the posterior cingulate and angular gyrus, without effects on the central executive system (Table 2).

**Table 2:** Neuroimaging studies of the acute effects of THC and cannabis on cognitive tasks.

Author	Imaging Modality	User Groups	Group Sample Size (n)	Group Definition	Drug	Task	User Age Mean (SD)	Dose of THC	Route	Increase (volume, blood flow, activation, connectivity)	Decrease (volume, blood flow, activation, connectivity)	Task Performance (THC vs comparison group or baseline)
Acute effects on attentional processing												
O'leary et al. (2000)	H_2_^15^0 PET	O	5	O = use <10 times/m for mean 3.2y	Cannabis	Auditory Attention Task	26.2 (8)	20mg	S	OFC, insula, temporal poles, ACC, cerebellum	Auditory cortex	No significant change
O'Leary et al. (2002)	H_2_^15^0 PET	O	12	O = use <10 times/m, mean 2.7 times/m	Cannabis	Auditory Attention Task	30.5 (8.6)	20mg	S	MPFC, insula, temporal poles, ACC, cerebellum	Auditory cortex, Visual cortex, Attentional Network (parietal, frontal, thalamus)	No significant change
O'leary et al. (2007)	H_2_^15^0 PET	O	12	O = use <10 times/m, mean 5.1 times/m, duration mean 3.1y	Cannabis	Auditory Attention Task	23.5 (4.3)	20mg	S	OFC, ACC, temporal pole, insula, cerebellum	Auditory cortex, Visual cortex	No significant change
Bhattacharyya et al. (2012)	fMRI	O	15	O = <15 uses per lifetime	THC	Visual Oddball task	26.7 (5.7)	10mg	PO	R PFC	R caudate	↓ reaction time
Bossong, van Hell, et al. (2013)	fMRI	O	20	O = mean 22.5 (SD 15.2) uses/last year, mean onset age 15.7 (SD 1.7), mean 7.3 (SD 5.1) years of use	THC	Continuous Performance Task	22.9 (4.9)	6mg	INH	PCC, angular gyrus	-	↑ false alarms, ↓ detected targets

Acute effects on response inhibition												
Borgwardt et al. (2008)	fMRI	O	15	O = <15 uses per lifetime	THC	Go/No-Go	26.7 (5.7)	10mg	PO	R HPC, R parahippocampal gyrus, R temporal cortex, L PCC	R ACC, R inferior frontal cortex	No significant change
Bhattacharyya et al. (2010)	fMRI	O	15	O = <5 uses per lifetime	THC	Go/No-Go	26.7 (5.7)	10mg	PO	Parahippocampal gyrus, L insula, L caudate	-	No significant change
Bhattacharyya et al. (2015)	fMRI	O	36	O = <25 uses per lifetime	THC	Go/No-Go	26.0 (5.5)	10mg	PO	-	L inferior frontal cortex	↑ inhibition errors, ↓ inhibition efficiency

Acute effects on reward function												
van Hell et al. (2012)	fMRI	O	14	O = ≥4 uses per year	THC	Monetary Incentive Delay	21.7 (2.3)	6mg	INH	-	Inferior parietal cortex, temporal cortex, PCC, ACC, OFC, R superior frontal cortex	No significant change
Jansma et al. (2013)	fMRI	Nicotine Addiction Group/C	10	Nicotine Addiction Group = mean 23.5 (SD 5.8) uses in last y; C = mean 22.6 (SD 3.6) uses in last y	THC	Monetary Incentive Delay	25.6 (2.1)	6mg	INH	-	NAc (Nicotine-Dependent Group)	No significant change
Freeman, Pope, et al. (2018)	fMRI	O	16	O = mean 8.06 (SD 5.5) uses/m, mean 8.94 (SD 7.0) years of use	Cannabis	Musical Reward	26.2 (7.3)	6% or 12%	INH	-	Auditory cortex, R HPC, R parahippocampal gurys, R amygdala, R ventral striatum	↑ Want to Listen to Music, ↑ Sound Perception

Acute effects on learning and memory												
Weinstein et al. (2008)	^18^F-FDG PET	Fr	12	Fr =≥1 use per day, ≥5 years of use, mean age of onset 19y, met DSM-IV criteria for dependence	THC	Virtual Reality Maze	27 (7.45)	17mg	S	Frontal cortex, ACC	Visual-Motor Areas	↑ Hitting the walls of the maze
Bhattacharyya et al. (2009)	fMRI	O	15	O = ≤15 uses per lifetime	THC	Verbal Paired Association Task	26.7	10mg	PO	Parahippocampal gyrus	Ventrostriatum	No significant change
Böcker et al. (2010).	EEG	O	16	O = r2-9 uses per month	Cannabis	Memory Search Task	Range 18-45	29.3mg, 49.1mg, or 69.4mg	S	-	Resting state theta power	↑ Errors, ↑Reaction time
Bossong, Jager, et al. (2012)	fMRI	O	14	O = mean 17.0 (SD 12.4) uses per year	THC	Sternberg Item Recognition	21.6 (2.1)	6mg	INH	Network-wide increase, cuneus, precuneus	R insula, R inferior frontal gyrus, L middle occipital gyrus	↓ Performance accuracy
Rabinak et al. (2014)	fMRI	O	14/14	O = <10 uses per lifetime	THC	Pavlovian Fear Extinction	Range 21-45	7.5mg	PO	VMPFC, HPC	-	No significant change
Bhattacharyya et al. (2018)	fMRI	O (TP/NP)	14/22	O = <25 uses per lifetime	THC	Verbal Learning Task	-	10mg	PO	L HPC (TP group)	-	No significant change

Acute effects on emotional processing												
Phan et al. (2008)	fMRI	O	16	O = mean 2.0 (SD 2.4) uses/m	THC	Angry/Fearful Face Matching	20.8 (2.6)	7.5mg	PO	-	Amygdala	No significant change
Fusar-Poli et al. (2009)	fMRI	O	15	O = <15 uses per lifetime	THC	Gender Discimination Task/Viewing Fearful Faces (Mild/Intense)	26.6 (5.7)	15mg	PO	R parietal lobe, L medial frontal gyrus (mild)/L precuneus, sensorimotor cortex (intense)	Middle-frontal gyrus, PCC (intense)	↑ SCR fluctuations
Bhattacharyya et al. (2010)	fMRI	O	15	O = <5 uses per lifetime	THC	Viewing Fearful Faces (Mild/Intense)	26.7 (5.7)	10mg	PO	Amygdala	L parahippocampal gyrus, R temporal cortex, occipital cortex	No significant change
Bossong, van Hell, et al. (2013)	fMRI	O	14	O = mean 20.0 (SD 9.4) uses/y	THC	Happy/Fearful Face Matching	21.5 (2.5)	6mg	INH	-	Amygdala-OFC-HPC-PFC-parietal cortex-occipital cortex network	↓ Performance accuracy during matching of fearful faces
Gorka et al. (2015)	fMRI	O	16	O = ≥10 uses per lifetime, <1 use/d	THC	Angry/Fearful Face Matching	20.8 (2.6)	7.5mg	PO	-	Amygdala-rostral ACC-MPFC network	No significant change
Gorka et al. (2016)	fMRI	O	41	O = <10 uses per lifetime	THC	Emotion Regulation Task (Passive experience of negative images – look, maintain, reappraise)	24.9 (3.8)	7.5mg	PO	Amygdala	Amygdala-DLPFC network	↓ negative affect following reappraise vs maintain condition, ↑ negative affect following maintain vs look, ↓ pleasant ratings and ↑ arousal ratings of unpleasant images

ACC = anterior cingulate cortex, C = control users, d = day, DLPFC = dorsolateral prefrontal cortex, DSM = Diagnostic & Statistic Manual of Mental Disorders, EEG = electroencephalogram, Fr = frequent cannabis users, F = female, FDG = fludeoxyglucose, fMRI = functional magnetic resonance imaging, HPC = hippocampus, INH = inhaled, L = left, m = month, M = male, MPFC = medial prefrontal cortex, NAc = nucleus accumbens, NP = transient psychotic symptoms not induced by THC, O = occasional cannabis users, OFC = orbitofrontal cortex, PFC = prefrontal cortex, PO = per os (oral), PET = positron emission tomography, r = range, R = right, S = smoked, SCR = skin conductance response, SD = standard deviation, THC = Δ^9^-tetrahydrocannabinol, TP = transient psychotic symptoms induced by THC, VMPFC = ventromedial prefrontal cortex, w = week, y = year.

### 3.4 Response inhibition

Using a Go/No-Go task 10mg oral THC increased the blood-oxygen-level dependent (BOLD) response in temporal and posterior regions yet attenuated responses in the anterior cingulate cortex (ACC) and inferior frontal cortices (Borgwardt et al., 2008). Studies using a similar task and dose (Bhattacharyya et al., 2010; Bhattacharyya et al., 2015) found that THC attenuated parahippocampal activation and inferior frontal activation, and the latter was inversely correlated with the frequency of inhibition errors and severity of psychotic symptoms. Vulnerability to inhibition errors is partially dependent on AKT1 genotype as A allele carriers of the rs1130233 single nucleotide polymorphism had increased inhibition errors compared to G allele homozygotes (Bhattacharyya et al., 2014). This may be clinically important as people who are more susceptible to the psychotogenic effects of cannabis are more likely to make inhibition errors than those who do not have a psychotogenic response (Atakan et al., 2013) and AKT1 genotype modulates risk of psychosis from cannabis use (Di Forti et al., 2012) and the acute psychotogenic effects of cannabis (Morgan, et al., 2016) (Table 2).

### 3.5 Reward function

Monetary reward tasks have been used to probe reward processing. Using the Monetary Incentive Delay (MID) task, inhaled THC (6mg using a vaporizer) induced a widespread attenuation of BOLD response to feedback in reward trials in the inferior parietal and temporal gyrus bilaterally, posterior and anterior cingulate, middle orbitofrontal gyrus, and right superior frontal gyrus (van Hell et al., 2012). An additional study by the same laboratory compared the effects of inhaled 6mg THC versus placebo in 11 healthy controls and 10 people with nicotine dependence (Jansma et al., 2013). THC did not influence response to reward feedback in healthy controls, consistent with the study by van Hell and colleagues (van Hell et al., 2012). However, THC reduced the NAc response to reward anticipation in nicotine-dependent participants. There is also evidence that cannabis influences other (non-monetary) rewards, such as music. Inhaled cannabis (containing THC but not CBD) dampened participants’ response to music reward in auditory cortex bilaterally and the right hemisphere hippocampus, parahippocampal gyrus, amygdala and ventral striatum (Freeman, Pope, et al., 2018). These effects were offset when participants were administered cannabis containing CBD as well as THC. This suggests that THC dampens the effects of consummatory rewards (consistent with van Hell et al., 2012), whereas CBD may offset this effect (Table 2).

### 3.6 Learning and memory

There is a high density of CB_1_Rs in the hippocampus and PFC (Curran et al., 2016) and disruptions of learning and memory are some of the most widely replicated acute effects of cannabis (Broyd et al., 2016). Using a Sternberg item recognition paradigm with four conditions (2–5 digits), THC caused a dose-dependent increase in reaction times and decrease in performance accuracy as a function of memory load (Böcker et al., 2010). This decline of working memory accuracy was significantly correlated with THC-induced decreases in resting state electroencephalography (EEG) theta power measured after task performance (Böcker et al., 2010). Bossong, Jansma, et al. (2012) studied the acute effects of THC inhalation (6 mg) on performance of a parametric Sternberg item recognition paradigm with five difficulty levels. During the placebo condition, brain activity increased linearly with rising working memory load. THC administration enhanced activity for low working memory loads, and reduced the linear relationship between working memory load and activity in a network of working memory related brain regions, and in left DLPFC, inferior temporal gyrus, inferior parietal gyrus, and cerebellum in particular. In addition, performance accuracy after THC was only reduced for moderately high working memory loads. These results suggest that participants exhibit enhanced brain activity during working memory tasks that they perform at normal level, indicating inefficient working memory function after THC administration (Bossong, Jansma, et al., 2012). Whilst no behavioural differences in recall tasks were observed during a verbal paired associative learning task, oral 10mg THC (vs. placebo) abolished the normal decrement in parahippocampal activation during encoding and attenuated ventrostriatal activation during word retrieval (Bhattacharyya et al., 2009). Under placebo conditions participants sensitive to the psychotogenic effects of cannabis had higher hippocampal activation during verbal encoding compared to participants without a psychotogenic response (Bhattacharyya et al., 2018). In keeping with these findings, while THC (6 mg inhaled) reduced activity during encoding in the right insula, the right inferior frontal gyrus, and the left middle occipital gyrus during performance of a pictorial associative memory task, activity during recall was significantly increased in a network of recall-related brain regions, with most prominent effects in the cuneus and precuneus. Although administration of THC did not affect performance accuracy, better performance was associated with lower recall activity during the placebo but not the THC condition (Bossong, Jager, et al., 2012). Using a Pavlovian fear extinction paradigm, pre-extinction acute THC (compared to placebo) caused increased ventromedial PFC and hippocampal activation to a previously extinguished conditioned stimulus during extinction memory recall (Rabinak et al., 2014). When users were administered oral THC (17mg) challenge while undergoing [^18^F]FDG PET and performing a virtual reality maze (Weinstein et al., 2008) acute THC caused more navigation errors and this was associated with increased metabolism in the frontal and anterior cingulate cortices (regions associated with motor coordination and attention), and reduced metabolism in areas that are related to visual integration of motion. Taken together these studies suggest that even when THC dose is not sufficiently high to result in deleterious effects on behavioural performance, increased brain activity has been reported across a range of tasks. One common interpretation of such results is that THC reduces the neural ‘efficiency’ of learning and memory processes. However, the term ‘efficiency’ in this context is problematic (Poldrack, 2015), and these results are consistent with a number of alternative explanations (Table 2).

### 3.7 Emotional processing

There is a high density of cannabinoid receptors in key areas of the brain involved in processing emotional stimuli, such as the amygdala and ACC (Herkenham et al., 1991; Katona et al., 2001). Moreover, the availability of CB_1_Rs receptor in the amygdala, assessed with PET imaging, seems to mediate the salience of threatening cues; particularly relevant to anxiety and salience processing in psychosis (Pietrzak et al., 2014).

Acute inhaled THC (8mg) impaired recognition of emotional faces at the behavioural level (Hindocha et al., 2015). Some studies also suggest that the effects of THC on emotional processing are valence specific. Using an emotional matching task, inhaled THC (6mg) impaired task performance, measured as mean percentage of correctly identified targets, for matching emotional faces with negative, but not positive emotional content (Bossong, van Hell, et al., 2013). In a network of brain regions including amygdala, orbitofrontal gyrus, hippocampus and PFC, neural activity was reduced while processing stimuli with a negative emotional content and increased during processing of positive stimuli. Using a similar paradigm, Phan et al. (2008) found that 7.5mg oral THC reduced amygdala reactivity to social signals of threat (angry and fearful faces) with no effect on response times, accuracy or subjective anxiety. This suggests that THC may play an anxiolytic role in fear behaviours. In a further analysis of the same data set, Gorka et al. (2015) showed that THC reduced functional coupling between the basolateral amygdala and superficial amygdala with the rostral ACC and medial PFC, respectively. It is possible that THC-induced hypoconnectivity between the amygdala and cortex underlies the dissociation between subjective and behavioural responses.

Two papers analysed data from a study using a gender discrimination task involving looking at mildly fearful and intensely fearful faces after 10mg oral THC in 15 healthy male volunteers. In the first paper, Fusar-Poli et al. (2009) found that THC increased skin conductance response amplitudes to fearful faces relative to both CBD and placebo. Also, THC primarily modulated activity in the frontal and parietal cortex to the faces, with no difference in the amygdala. Specifically, during processing of mildly fearful faces, THC increased activation in the right inferior parietal lobule, and decreased activation in the left medial frontal gyrus. Activity in the left precuneus and primary sensorimotor cortex increased during processing related to intensely fearful faces, with decreased activation seen in the middle frontal gyrus and posterior cingulate gyrus. During the processing of fearful faces (mild plus intense) THC decreased activation in the right inferior frontal gyrus, right superior temporal gyrus, and left medial frontal gyrus, and increased activation in the left precuneus. This suggests that THC-induced anxiogenesis may not be mediated through amygdala reactivity. In a subsequent paper, Bhattacharyya et al. (2010) investigated areas where CBD and THC had opposite effects, which included the cerebellum, fusiform gyrus, lingual gyrus, lateral PFC and the amygdala. These opposite effects of THC and CBD are consistent with evidence that THC and CBD have opposite effects on emotional face recognition at the behavioural level, and that CBD can protect against THC-induced impairments in face recognition (Hindocha, et al., 2015).

Further evidence of THC-induced increases in amygdalar response during implicit and explicit emotional processing comes from research using the International Affective Picture System (Gorka, et al., 2016). Compared to placebo, 7.5mg THC resulted in increased left amygdala activation during the passive experience of unpleasant images compared to looking at neutral images. This suggests that amygdala activation to negative stimuli is greater after a THC challenge. Furthermore, the THC group exhibited greater left amygdala activation, and less amygdala-DLPFC coupling during cognitive reappraisal, in comparison to placebo.

These studies indicate that THC has complex effects on BOLD responses to fearful faces, involving a pattern of increased and decreased activation in both frontal and parietal areas. Although both studies (Bossong, van Hell, et al., 2013; Fusar-Poli et al., 2009) found lower THC-induced brain activity in prefrontal and temporal areas during processing of threatening stimuli, differences in the results (Bossong, van Hell, et al., 2013; Fusar-Poli et al., 2009; Phan et al., 2008) may reflect differences in the functional magnetic resonance imaging (fMRI) task. In contrast to the other two studies, Fusar-Poli et al. (2009) used a gender discrimination task, which did not require explicit processing of the emotional content of the stimuli. In a further exploration of this fMRI study on emotional processing, Fusar-Poli et al. (2010) did not show any effects of THC administration on connectivity between the amygdala and ACC. Nonetheless, all studies suggest a striking difference between the acute effects of THC on processing of emotions and on experiencing of emotions. Whereas THC shifts the emotional bias away from fearful stimuli in most studies (Bossong, van Hell, et al., 2013; Fusar-Poli et al., 2009; Phan et al., 2008) its administration enhances subjective feelings of anxiety, particularly when high doses are given to less experienced participants in a laboratory setting (Crippa et al., 2009; D'Souza et al., 2004; Ilan et al., 2005; Karniol et al., 1974; Morrison et al., 2009; Zuardi et al., 1982) (for a review see Crippa et al. (2009)) (Table 2).

### 3.8 The dopaminergic system

PET can directly measure the dopaminergic system using radiolabelled selective dopamine receptor antagonists such as [^11^C]-raclopride. Using PET and the dopamine D_2/3_ receptor tracer [^11^C]-raclopride in seven healthy volunteers, Bossong et al. (2009) found that inhalation of THC (8 mg) induced a moderate but significant reduction in [^11^C]-raclopride binding in the ventral striatum and precommissural dorsal putamen (3.4% and 3.9%, respectively), which is consistent with an increase in dopamine levels in these regions (Bossong et al., 2009). Stokes et al. (2009) scanned thirteen healthy subjects using a similar PET methodology, but did not show effects of oral THC administration (10 mg) on [^11^C]-raclopride binding, despite an increase in schizophrenia-like symptoms. However, although not statistically significant, THC administration caused a radiotracer displacement of 1.6% and 3.2% in the right and left ventral striatum, respectively, which is within a similar range to that reported by Bossong et al. (Stokes et al., 2009). A pooled re-analysis of these two studies revealed a significant reduction in [^11^C]-raclopride binding in the limbic striatum (−3.65%) after THC administration (Bossong et al., 2015). Finally, using single photon emission computerized tomography and [^123^I]-iodobenzamide, Barkus et al. (2011) failed to show an effect of intravenously administered THC (2.5 mg) on striatal dopamine concentrations in nine healthy men. Unfortunately, this study was not conducted at radiotracer equilibrium conditions, thus not allowing quantifiable information regarding the effects of the challenge. Collectively, these data provide human evidence for a modest increase in striatal dopamine transmission after administration of THC compared to other drugs of abuse.

### 3.9 Interactions with γ-aminobutyric acid (GABA)

Using EEG, Radhakrishnan et al. (2015) used pre-treatment with iomazenil, an iodine analogue of the benzodiapine receptor competitive antagonist flumazenil, to demonstrate that GABA deficits enhance the neuropsychopharmacological effects of intravenous THC (1.05mg/kg). When pre-treated with iomazenil, THC induced significantly greater psychotic symptoms, perceptual alterations, subjective distress and a concomitant reduction in THC-induced P300 amplitude. This may be clinically important because reductions in P300 amplitude have been observed in psychiatric illnesses including schizophrenia (Bramon et al., 2004).

## 4 The chronic effects of cannabis and THC

### 4.1 Whole brain volume

Early studies used computed tomography (CT) to investigate whether cannabis use was associated with structural alterations in the brain and found that cannabis users did not exhibit gross atrophic changes (Co et al., 1977; Hannerz & Hindmarsh, 1983; Kuehnle et al., 1977). However, early CT suffered from having limited volumetric data from soft tissue. Since then, no study has reported significant differences in whole brain volume between cannabis users and controls, although differences have been reported when cortical grey and white matter are examined separately (Lorenzetti et al., 2010). One study (Wilson et al., 2000) found that early cannabis exposure was associated with decreased grey matter volume and increased white matter volume in early onset users, although this was not replicated by another study (Tzilos et al., 2005).

### 4.2 Regional brain structure

As per initial CT research, early MRI studies did not find significant structural deficits associated with cannabis use (Block, O'Leary, Ehrhardt, et al., 2000; Jager et al., 2007; Tzilos et al., 2005). Subsequently, hippocampal and parahippocampal atrophy have been associated with chronic cannabis use (Ashtari et al., 2011; Demirakca et al., 2011; Filbey et al., 2015; Lorenzetti et al., 2015; Matochik et al., 2005; Yücel et al., 2008). Even in studies that did not find significant reductions in users compared to non-users, there was evidence of a negative correlation between cannabis exposure and dependence severity with hippocampal volume (Chye et al., 2018; Cousijn et al., 2012). Since the lack of regional effects may be influenced by lateralisation, a meta-analysis found that when the left and right hippocampi are combined there was evidence of hippocampal reduction (Rocchetti et al., 2013). However, a longitudinal study of hippocampal volume in heavy cannabis users (mean age 21 years) compared to non-users (Koenders et al., 2016; Koenders et al., 2017) did not find cannabis-induced effects at baseline or 39-month follow-up using voxel-based and manual tracing approaches. This is consistent with another, recent study using voxel-based analysis, which also revealed no structural changes to the hippocampal volume in chronic users (Moreno-Alcazar et al., 2018). Nonetheless, inconsistencies may be due to dependence and/or specific effects within the hippocampus as other recent work has found that volume deficits are most prominent in the cornu ammonis 1-3 subfields and dentate gyrus in cannabis-dependent users (Chye, Suo, et al., 2017). This would tie in with previous findings that cannabis use disorder was associated with morphological differences within the hippocampus that were related to episodic memory impairments (Smith, Cobia, et al., 2015). Atrophic and dysmorphogenic effects of cannabis on subcortical structures have been extended to the amygdala and NAc (Lorenzetti et al., 2015; Yücel et al., 2008), and hypertrophic changes have also been described in the basal ganglia of cannabis users (Moreno-Alcazar et al., 2018). In terms of cortical regions, heavy cannabis users have abnormal gyrification (type III), reduced orbitofrontal volume (Chye, Solowij, et al., 2017) and reduced right anterior cingulate volume compared to non-users, which is influenced by CB_1_R haplotype variation (Hill et al., 2016) (Table 3).

**Table 3:** Neuroimaging studies of the chronic effects of cannabis on brain structure and volume

Author	Imaging Modality	Users/Controls (n)	User age, mean (SD) unless otherwise stated	Duration of use (y), mean (SD) unless otherwise stated	User onset age (y), mean (SD) unless otherwise stated	Use frequency in joints/cones/uses, mean (SD) unless otherwise specified	Increase (volume, blood flow, activation, connectivity)	Decrease (volume, blood flow, activation, connectivity)
Chronic effects on whole brain structural volume								
Co et al. (1977)	CT	12/34	24.1 (-)	6.6 (-)	17.4 (-)	9 (-)/d	No significant changes	
Kuehnle et al. (1977)	CT	19/19	23.8 (-)	Inpatient ward study (21d)	-	34.7 (-)/m	No significant changes	
Hannerz and Hindmarsh (1983)	CT	12/12	26.1 (-)	10.25 (-)	-	-	No significant changes	
Wilson et al. (2000)	sMRI & H_2_^15^0 PET	57/0	31.3 (7)	16.9 (6.4) early onset [<17yo] males and females 13.4 (6.0), late onset [>17yo] males 13.9 (6.9) and females 14.0 (6.6)	16.8 (3.6)	240.8 (198.1) early onset [<17yo] males and females 146.5 (128.7), late onset [>17yo] males 205.6 (587.0) and females 128.2 (186.8)/y	WM volume (early-onset [<17y] users only)	GM volume, whole brain (early onset users [<17yo] only)
Block, O'Leary, Ehrhardt, et al. (2000)	sMRI	18/13	22.3 (0.5)	3.9 (0.4)	-	18 (2)/w	-	Ventricles
Tzilos et al. (2005)	sMRI	22/26	38.1 (6.2)	22.6 (5.7)	16 (4.0)	≧1/d	No significant changes	
Jager et al. (2007)	sMRI	20/20	24.5 (5.2)	-	-	322.5 (-)/y	No significant changes	

Chronic effects on regional brain structure								
Block, O'Leary, Ehrhardt, et al. (2000)	sMRI	18/13	22.3 (0.5)	3.9 (0.4)	-	18 (2)/w	No significant changes	
Matochik et al. (2005)	sMRI	11/8	29.7 (4.7)	7.5 (5.5)	15.7 (2.5)	34.7 (17.6)/w	Precuneus, thalamus, parahippocampal gyrus, pons, lentiform nucleus, fusiform gyrus.	HPC GM, R parahippocampal GM, L parietal WM.
Tzilos et al. (2005)	sMRI	22/26	38.1 (6.2)	22.6 (5.7)	16 (4.0)	≧1/d	No significant changes	
Jager et al. (2007)	sMRI	20/20	24.5 (5.2)	-	-	322.5 (-)/y	No significant changes	
Yücel et al. (2008)	sMRI	15/16	39.8 (8.9)	39.8 (8.9)	20.1 (6.9)	28 (4.6)/m	-	HPC, amygdala
Ashtari et al. (2011)	sMRI	14/14	19.3 (0.8)	-	13.1 (-)	5.8 (-)/d	-	HPC (note 6.7m abstinent before trial)
Demirakca et al. (2011)	sMRI	11/13	r19-25	5.4 (-)	-	-	-	R anterior HPC
Cousijn et al. (2012)	sMRI	33/42	21.3 (2.4)	2.5 (1.9)	18.8 (2.3)	4.9 (1.5)/w	Anterior Cerebellum	HPC, amygdala (correlates with amount of cannabis use)
Filbey et al. (2014)	sMRI & fMRI	48/62	28.3 (8.3)	9.8 (8.0)	18.1 (3.4)	11.1 (1.4)/w	OFC-Forceps Minor Network Connectivity	Orbifrontal gyrus volume
Filbey et al. (2015)	sMRI	36 (cannabis users)/19 (nicotine users)/19 (cannabis + nicotine users)/16 (controls)	24.9 (8.8) [cannabis users], 23.3 (7.3) [cannabis + nicotine users]	-	-	80.6 (14.2)/last 90d [cannabis users], 82.2 (11.5)/last 90d [cannabis + nicotine users]	-	HPC (cannabis users and cannabis + nicotine users)
Lorenzetti et al. (2015)	sMRI	15/16	40 (9)	21 (-)	-	28 (3)/m	-	HPC, amygdala
Smith, Cobia, et al. (2015)	sMRI	10 (cannabis users)/28 (SZP)/15 (SZP + cannabis users)/44 (controls)	-	2.6 (2.5)	16.7 (-)	80% were daily users	Altered HPC morphology (cannabis users and SZP+cannabis users vs. controls)	
Hill et al. (2016)	sMRI	34 (split into lower/higher cannabis use groups)/54	27.2 (4.3) [lower use], 26.4 (2.8) [higher use]	3.0 (2.9) [lower use], 6.3 (3.1) [higher use]	18.1 (4.4) [lower use], 18.5 (-) [higher use]	9,167.9 (16,770.9) [lower], 17,756.2 (21,036.3) [higher]/lifetime	-	R anterior cingulate (associated with CNR1 haplotype variation)
Koenders et al. (2016)	sMRI	20/22 baseline, 39m	20.5 (2.1)	-	14.5 (1.65)	4.7 (1.6) [baseline], 2.9 (2.3) [39m]/w	No significant changes	
Koenders et al. (2017)	sMRI	20/23 baseline, 39m	20.6 (2.2)	-	16.1 (2.3)	4.7 (1.6) [baseline], 5.1 (2.3) [39m follow-up]/w	No significant changes	
Chye, Solowij, et al. (2017)	sMRI	22 ND/39 D/35 controls	36.2 (11.7) [ND], 30.3 (10.0) [D]	-	17.2 (3.2) [ND], 16.4 (3.4) [D]	21.9 (10.3) [ND], 27.4 (4.5) [D]/m	-	CA1, CA2, CA3, CA4/dentate gyrus, total HPC GM
Chye, Suo, et al. (2017)	sMRI	140/121	28.0 (10.2)	-	17.8 (3.3)	334.1 (322.3)/m	No significant changes in users vs control; medial-lateral OFC (D vs ND only, F>M)	
Chye et al. (2018)	sMRI	1: 140 [cannabis users]/121 [controls] 2: 50 [ND]/70 [D]/106 [controls] 3: 41 [ND]/41 [D]/41 [controls]	1: 28.0 (10.3) [cannabis users], 2: 27.1 (7.3) [ND], 26.7 (9.2) [D], 3: 28.6 (10.8) [ND], 26.7 (8.5) [D]	-	1: 17.8 (3.4) [cannabis users], 2: 17.8 (2.7) [ND], 17.4 (3.4) [D], 3: 17.8 (2.8) [ND], 17.5 (2.6) [D]	1: 334.1 (322.3)/m [cannabis users], 2: 229.8 (202.3)/m [ND], 351.6 (291.0)/m [D], 3: 235.4 (209.9)/m [ND], 278.9 (172.8)/m [D]		HPC volume [D only]
Moreno-Alcazar et al. (2018)	sMRI	14/28 (control group 1)/100 (control group 2)	30.1 (5.2)	14.4 (6.7)	17.1 (2.1)	8.4 (3.8)/d	GM cluster in basal ganglia (caudate, putamen, pallidum, NAc); larger volume in putamen, pallidum	-

CA = cornu ammonis, CNR1 = cannabinoid receptor 1 gene, CT = computed tomography, d = day, D = dependent cannabis user, F = female, fMRI = functional magnetic resonance imaging, GM = gray matter, HPC = hippocampus, L = left, m = month, M = male, NAc = nucleus accumbens, ND = non-dependent cannabis user, OFC = orbitofrontal cortex, PET = positron emission tomography, r = range, R = right, SD = standard deviation, sMRI = structural magnetic resonance imaging, SZP = schizophrenia, w = week, WM = white matter, y = year.

### 4.3 Structural connectivity

One of the three early diffusion tensor imaging studies found evidence of structural dysconnectivity in cannabis users (Arnone et al., 2008; Delisi et al., 2006; Gruber & Yurgelun-Todd, 2005) in the form of reduced mean diffusivity in the prefrontal section of the corpus callosum. Chronic cannabis users were later found to also have microstructural dysconnectivity in the splenium of the corpus callosum, fornix and commissural fibres (Zalesky et al., 2012). Applying graph theory to diffusion tensor imaging and tractography, Kim, et al. (2011) found that cannabis users had less efficiently integrated global structural networks alongside altered local connectivity in the cingulate. There is also evidence from a small study that reduced frontal white matter connectivity was associated with impulsivity in cannabis users (Gruber et al., 2011), however since impulsivity is a risk factor for drug use it is possible that this pre-dates the cannabis use. Nonetheless, other studies have found effects on orbitofrontal connectivity whereby structural fractional anisotropy in the forceps minor increased with regular use but then decreased following long-term heavy use (Filbey et al., 2014), which would support an effect of drug use on structural connectivity.

The first longitudinal evidence for cannabis effects on white matter structure came from two studies (Becker et al., 2015; Epstein & Kumra, 2015). Compared to controls, adolescents with cannabis use disorder had reduced connectivity in the left inferior longitudinal fasciculus (Epstein & Kumra, 2015) while cannabis using young adults had attenuated growth in white matter connectivity in several key pathways (Becker et al., 2015). Importantly, greater cannabis consumption was associated with reduced connectivity. These findings were corroborated by a large study of 466 adults reporting recreational cannabis use from the Human Connectome Project (Orr et al., 2016). Whilst that study did not find group differences between recreational users and non-users, there was a relationship between age of onset of cannabis use and reduction in white matter coherence in tracts reported previously including the superior and inferior longitudinal fasciculi, and the major and minor forceps of the corpus callosum connecting the left and right occipital and frontal lobes, respectively (Table 4).

**Table 4:** Neuroimaging studies of the chronic effects of cannabis on structural connectivity

Author	Imaging Modality	Users/Controls (n)	User age, mean (SD) unless otherwise stated	Duration of use (y), mean (SD) unless otherwise stated	User onset age (y), mean (SD) unless otherwise stated	Use frequency in joints/cones/uses, mean (SD) unless otherwise specified	Increase (volume, blood flow, activation, connectivity)	Decrease (volume, blood flow, activation, connectivity)
Chronic effects on structural connectivity								
Gruber & Yurgelun-Todd (2005)	DTI	10/10	26.8 (3.6)	-	14.1 (-)	39.4/w	No significant changes	
Delisi et al. (2006)	DTI	10/10	23.0 (4.4)	>1y	<18	r:1/d to 3/w	No significant changes	
Arnone et al. (2008)	DTI	11/11	25.0 (2.9)	9.0 (3.5)	15.2 (2.8)	44.1 (29.4)/w	Corpus Callosum (Mean Diffusivity)	-
Kim et al. (2011)	DTI (with graph theory)	12/13	19.3 (0.9)	3.3 (2.5)	16.0 (2.3)	5 (1.7)/w	Clustering Coefficients	Global network efficiency/Altered cingulate connectivity
Zalesky et al. (2012)	DW-MRI	59/33	33.4 (10.9)	15.6 (9.5)	16.7 (3.3)	147 (142)/m	-	R fimbria of HPC (fornix), splenium of corpus callosum, commissural fibres [changes associated with age of onset use]
Gruber et al. (2011)	DTI	15/15	25.0 (8.7)	10.1 (9.7)	14.9 (2.5)	25.5(27.8)/w	R Genu (Higher trace)	L Frontal (FA)
Filbey & Dunlop (2014)	DTI	31 D/24 ND	24.4 (6.9) [D]/24.4 (8.0) [ND]	5.8 (5.8) [D]/7.6 (7.8) [ND]	18.1 (3.6) [D]/17.0 (2.6) [ND]	80.8 (14.3) [D]/82.5 (14.8) [ND]/last 90d	Amygdala-ACG [D] connectivity, NAc-OFC-HPC [ND] connectivity	-
Becker et al. (2015)	DTI	23/0 baseline, 2y	19.5 (0.7)	>1y	15.4 (1.2)	3032.6 (2395.3)/last y [baseline]	-	Growth of superior longitudinal fasciculus, L superior frontal WM, L corticospinal tract, R anterior thalamic radiation (FA) R central/posterior superior longitudinal fasciculus, corticospinal tract, posterior cingulum (diffusion)
Epstein & Kumra (2015)	DTI	19 [D]/34 EOSS (occasional cannabis users)/29 controls baseline, 18m	16.6 (1.5)	-	<17	712 (399) d/lifetime	-	L inferior longitudinal fasciculus, L inferior-fronto-occipital fasciculus (FA)
Orr et al. (2016)	DTI & sMRI	466 (Human Connectome Project)	r22-35	-	r <14 to >21	r 1-5/lifetime to >1000/lifetime	-	WM coherence in superior & inferior longitudinal fasciculus, corpus callosum (major & minor forceps, occipital & frontal lobe levels); changes correlate with age of onset of cannabis use only - no group differences in cannabis users vs non-users.

ACG = anterior cingulate gyrus, d = day, D = dependent cannabis user, DTI = diffusion tensor imaging, DW-MRI = diffusion-weighted magnetic resonance imaging, EOSS = early-onset schizophrenia spectrum disorder, FA = fractional anisotropy, fMRI = functional magnetic resonance imaging, HPC = hippocampus, L = left, m = month, NAc = nucleus accumbens, ND = non-dependent cannabis user, OFC = orbitofrontal cortex, r = range, R = right, SD = standard deviation, sMRI = structural magnetic resonance imaging, w = week, WM = white matter, y = year.

### 4.4 Resting cerebral blood flow and metabolism

A range of neuroimaging techniques have been used to measure the long-term effects of THC on CBF including [^133^Xe] cerebral blood flow tomography, H_2_[^15^O]- PET, single-photon emission computed tomography, fMRI and ASL. [^133^Xe] inhalation comparing CBF in long-term cannabis users after cessation compared to controls has found lower global (Tunving et al., 1986) and frontal (Lundqvist et al., 2001) CBF, although this has not been replicated in all studies (Mathew et al., 1986). In contrast to findings in “inexperienced users”, [^133^Xe] imaging found that there was no significant effect of acute inhaled cannabis on CBF in “experienced users” relative to placebo (Mathew et al., 1989). More recent studies using ASL (Jacobus et al., 2012) have found that cannabis users have reduced CBF in the left superior and middle temporal gyri, left insula, medial frontal gyri and left supramarginal gyrus alongside increased CBF in the right precuneus. Studies using H_2_ [^15^O]-PET have found reductions of 18% in regional CBF in ventral PFC and bilateral posterior cerebellar hemisphere in “frequent” cannabis users, compared to controls after 26 hours of abstinence (Block, O'Leary, Hichwa, et al., 2000). Paradoxically, in one study (Wilson et al., 2000) earlier age of first cannabis use was associated with relatively higher global CBF compared to those who started later. More novel MRI methods including dynamic susceptibility contrast MRI and phase contrast MRI have yielded conflicting results including increased blood volume in the right frontal and temporal cortices and cerebellum (Sneider et al., 2008) in users, which were not present upon four weeks cessation, and increased striatal CBF (Filbey et al., 2018).

A limited number of studies have investigated brain metabolism in cannabis users with [^18^F]FDG PET. Wiers et al. (2016) found that people with cannabis use disorder had frontal hypometabolism, including in the anterior cingulate, which was associated with negative emotionality. Upon methylphenidate challenge cannabis users had an attenuated whole-brain glucose metabolic response with the most pronounced effects in the striatum. Within cannabis users methylphenidate-induced metabolic increases in the putamen were inversely related with addiction severity. Of note, there were significant sex effects, such that both the group differences at baseline in frontal metabolism and the attenuated regional brain metabolic responses to methylphenidate were observed in female but not male users. The hypofrontality findings above are in line with those of one previous study which found that cannabis users had hypometabolism in the OFC, precuneus and putamen (Sevy et al., 2008). Importantly, there was no relationship between dopamine receptor availability and glucose metabolism (Sevy et al., 2008) (Table 5).

**Table 5:** Neuroimaging studies of the chronic effects of cannabis on cerebral blood flow and metabolism, and functional connectivity.

Author	Imaging Modality	Users/Controls (n) unless otherwise stated	Pre-trial abstinence, mean days (SD) unless otherwise stated	Activity	Mean User Age (SD)	Duration of use, mean years (SD)	Use onset age (SD)	Use frequency in joints/cones/uses, mean (SD) unless otherwise stated	Increase (volume, blood flow, activation, connectivity)	Decrease (volume, blood flow, activation, connectivity)	Task Performance (cannabis user vs comparison group)
Chronic changes on cerebral blood flow and metabolism											
Mathew et al. (1986)	^133^Xe SPECT	17/16	0.5 (-)	Resting	25.5 (8)	6.9 (-)	-	14.0 (-)/w	No Significant Changes		-
Tunving et al. (1986)	^133^Xe SPECT	9 [cannabis users]/4 [users re-examined after further abstinence]/0 controls	r1-12 (n=9)/r9-60 (n=4)	Resting	24.2 (-)	9.8 (-)	-	6.7 (-)/w	Frontal (cannabis users at follow-up after abstinence)	Global CBF	-
Block, O'Leary, Hichwa, et al. (2000)	H_2_^15^0 PET	17/12	1.3 (0.0)	Resting	22.4 (0.5)	3.9 (0.5)	-	≧7 (3)/w	-	VPFC, posterior cerebellar hemisphere	-
Wilson et al. (2000)	sMRI & H_2_^15^0 PET	57/0	14 (-)	Resting	31.3 (7)	early onset [<17yo] 16.9 (6.4) [M] and 13.4 (6.0) [F], late onset [>17yo] 13.9 (6.9) [M] and 14.0 (6.6) [F]	16.8 (3.6)	early onset [<17yo] 240.8 (198.1) [M] and 146.5 (128.7) [F], late onset [>17yo] 205.6 (587.0) [M] and 128.2 (186.8) [F]/y	Global (in early onset [<17yo] vs late onset [>17yo])	-	-
Lundqvist et al. (2001)	^133^Xe SPECT	14/14	1.6 (-)	Resting	29.8 (5.0)	8.3 (5.6)	-	2.4 (1.7) grams/day	-	Frontal, Global	-
Sevy et al. (2008)	^18^F-FDG PET	6/6	60 (20)	Resting	20.1 (1)	7.0 (1.0)	12.0 (2.0)	16.0 (12.0) grams/day	-	R OFC, putamen, precueneus	-
Sneider et al. (2008)	DSC-MRI	15/17	0/7/28 [longitudinal study over 28 days of abstinence]	Resting	38.3 (5.6) - note users significantly older than controls	-	-	20,601.3 (13,540.8)/lifetime	R frontal, L temporal, cerebellum (day 0)/R frontal, temporal, cerebellum (day 7, M>F)/L temporal area, cerebellum (day 28)	-	-
Jacobus et al. (2012)	ASL	23/23	5.1 (3.8)	Resting	17.7 (0.7)	-	-	17.9 (9.2)/m	R precuneus	L superior and middle temporal gyri, L insula, medial frontal gyri, L supramarginal gyrus	-
Wiers et al. (2016)	^18^F-FDG PET	24/24	-	Resting	29.0 (8.8) [M], 24.6 (4.3) [F]	12.9 (9.1) [M], 9.0 (4.7) [F]	14.8 (3.0) [M], 15.2 (2.4) [F]	4.9 (3.8)/d [M], 4.8 (2.9)/d [F]	-	Frontal including ACC (F only), striatum (post-methyphenidate challenge)	-
Filbey et al. (2018)	TOFA, PC-MRI, TRUST-MRI, ASL	74/101	3.3 (0.4)	Resting	31.3 (7.9)	10.6 (7.3)	-	14,173.8 (10,866.0)/lifetime	Global OEF and CMRO_2_, R pallidum/putamen, global CBF & R superior frontal cortex (positively correlated with serum THC levels)	-	-

Chronic effects on functional connectivity											
Filbey & Dunlop (2014)	DTI	31 [D]/24 [ND]	-	Resting	24.4 (6.9) [D]/24.4 (8.0) [ND]	5.8 (5.8) [D]/7.6 (7.8) [ND]	18.1 (3.6) [D]/17.0 (2.6) [ND]	80.8 (14.3) [D]/82.5 (14.8) [ND]/last 90d	Amygdala-ACG connectivity [D]/NAc-OFC-HPC connectivity [ND]	-	-
Cheng et al. (2014)	fMRI	12/13	>0.5	Resting	19.3 (1.0)	3.3 (2.4)	16.0 (2.3)	12.8 (10.9)/w	Increase resting state in diffuse regions (expressing CB1R)	-	-
Pujol et al. (2014)	fMRI	28/29	31 (-)	Resting	21.0 (2.0)	6.0 (2.5)	14.9 (1.0)	899 (560)/y	Ventral PCC	Dorsal PCC-precuneus, HPC (related to memory impairments)	-
Lopez-Larson et al. (2015)	fMRI	43/31	No abstinence	Resting	18.0 (1.2)	-	14.7 (1.4)	14.8 (15.0)/w	OFC-PFC-ACC	-	-
Lichenstein et al. (2017)	fMRI	29 (divided into stable-high use [A], escalating use [B], stable-low use [C])	-	Resting	20.0 (0.0)	-	15.7 (2.0)	9.5 (12.2)/m	NAc-MPFC [A/C]	NAc-MPFC [B]	-
Manza et al. (2018)	fMRI	30/30 [Human Connectome Project]	-	Resting	29.2 (3.1)	-	-	-	Ventral striatum, Midbrain, Brainstem, Lateral thalamus	-	-

A = stable-high use, ACC = anterior cingulate cortex, ACG = anterior cingulate gyrus, ASL = arterial spin labelling, B = escalating use, C = stable-low use, CB1R = endocannabinoid 1 receptor, CBF = cerebral blood flow, CMRO_2_ = Cerebral Metabolic Rate of Oxygen, d = day, D = dependent users, DTI = diffusion tensor imaging, F = female, fMRI = functional magnetic resonance imaging, FDG = fludeoxyglucose, h = hour, HPC = hippocampus, L = left, m = month, M =male, MPFC = medial prefrontal cortex, NAc = nucleus accumbens, ND = non-dependent users, OEF = Oxygen Extraction Fraction, OFC = orbitofrontal cortex, PCC = posterior cingulate cortex, PC-MRI = phase contrast magnetic resonance imaging, PET = positron emission tomography, PFC = prefrontal cortex, r = range, R = right, SPECT = single photon emission computed tomography, SD = standard deviation, THC = Δ^9^-tetrahydrocannabinol, TOFA = time of flight angiogram, TRUST-MRI = T2 relaxation under spin tagging magnetic resonance imaging, VPFC = ventral prefrontal cortex, w = week, y = year.

### 4.5 Functional connectivity

Long-term cannabis use is associated with a range of functional connectivity alterations. Cannabis abuse and dependence have also been associated with increased local functional connectivity in the ventral striatum and midbrain (Manza et al., 2018) alongside striatofrontal hypoconnectivity (Filbey & Dunlop, 2014; Lichenstein et al., 2017). This is associated with escalating patterns of use, anhedonia and lower educational achievement at age 22 years (Lichenstein et al., 2017). In addition, cannabis users showed increased functional connectivity in the ventral part of the posterior cingulate cortex (PCC) and decreased functional connectivity in the dorsal PCC-precuneus junction alongside hippocampal hypoconnectivity such that aberrant default mode and hippocampal connectivity were related to memory impairments (Pujol et al., 2014). Compared to controls, male cannabis users had increased resting state activity in diffuse regions corresponding to those with high CB_1_R expression (Cheng et al., 2014). Increased functional connectivity observed between these regions and increased resting state activity was related to impulsivity. In line with structural hyperconnectivity of the OFC seen in young cannabis users described above there is evidence that users have increased functional connectivity in the OFC and the minor forceps which was associated with age of onset of drug use (Filbey & Dunlop, 2014). This finding was replicated in a separate study using seeds in the OFC (Lopez-Larson et al., 2015) whereby increased orbitofrontal connectivity with the PFC and ACC was observed in adolescent heavy cannabis users (Lopez-Larson et al., 2015). Importantly, this was related to both cannabis use and impulsivity (Table 5).

### 4.6 Executive function

Cannabis use is associated with executive dysfunction. Using the Iowa Gambling Task (Bechara et al., 1994) and H_2_[^15^O]- PET (Bolla et al., 2005; Vaidya et al., 2012) there is evidence, including dose-effects, that chronic cannabis users have prefrontal dysfunction. These findings were extended using fMRI whereby heavy cannabis users had hyperactivation to win versus loss evaluation in the right OFC, right insula, and left superior temporal gyrus compared to non-users (Cousijn et al., 2013). One study (Gruber et al., 2017) examined the effects of three months exposure to “medical” cannabis. While that study reported improved task performance and purported normalisation of aberrant BOLD response, the clinical groups were heterogeneous, there was no placebo group, and the doses of phytocannabinoids were not reported, which limits the inferences that can be made.

Cannabis users exhibit deficits in attention, however there are conflicting findings in the neuroimaging literature regarding underlying mechanisms. For example, both increases and decreases in right PFC function have been reported (Abdullaev et al., 2010; Chang, Yakupov, et al., 2006) as well as no significant effects (Jager et al., 2006). In a study of the interactions between attention-deficit hyperactivity disorder and cannabis use with a prospective cohort (Kelly et al., 2017) there were distinct effects of diagnosis and cannabis use on network connectivity. Importantly, that study did not report cannabis-associated exacerbations of impaired network connectivity, which were found in patients with attention-deficit hyperactivity disorder. However, this may be due to cannabis users who were regular but not daily users. Taken together there is evidence that disrupted executive network function may underlie the behavioural attentional deficits seen in cannabis use.

In terms of response inhibition, there is electrophysiological evidence from a drug Stroop task that cannabis users have an enhanced early attentional bias to drug-related cues (Asmaro et al., 2014). Using the Stroop and Go/No-go tasks, cannabis users have impaired response inhibition compared to non-users (Gruber & Yurgelun-Todd, 2005; Hester et al., 2009) associated with anterior cingulate hypoactivation, which has also been reported in the absence of behavioural differences in performance (Eldreth, Matochik, Cadet, & Bolla, 2004). In terms of connectivity, Go/No-go and stop-signal experiments (Behan et al., 2014; Filbey & Yezhuvath, 2013) found that poor inhibitory control in cannabis users was related to parieto-cerebellar hyperconnectivity and cannabis dependence was associated with fronto-nigro-subthalamic hyperconnectivity during successful response inhibition.

There is converging evidence that cannabis use is associated with working memory impairments associated with hyperactivation and hyperconnectivity of working memory circuits particularly in the PFC (Becker et al., 2010a; Colizzi et al., 2015; Jager et al., 2010; Kanayama et al., 2004; Tervo-Clemmens et al., 2018). These effects have been associated with total cannabis exposure (Tervo-Clemmens et al., 2018) which may be mediated by CB_1_R genotype (Colizzi et al., 2015). Whilst a study in chronic heavy users did not find a significant difference between cannabis users and controls, there was a disturbance of the normal relationship between performance improvement and concomitant changes in network function (Cousijn et al., 2013). Working memory effects may predict severity of subsequent drug use (Cousijn et al., 2014). However, these effects do not appear to persist into abstinence (Jager et al., 2006) (Table 6).

**Table 6:** Neuroimaging studies of the chronic effects of cannabis on executive function and motor performance.

Author	Imaging Modality	Users/Controls (n) unless otherwise stated	Pre-trial abstinence, mean days (SD) unless otherwise stated	Activity	Mean User Age (SD)	Duration of use, mean years (SD)	Use onset age (SD)	Use frequency in joints/cones/uses, mean (SD) unless otherwise stated	Increase (volume, blood flow, activation, connectivity)	Decrease (volume, blood flow, activation, connectivity)	Task Performance (cannabis user vs comparison group)
Chronic effects on executive function											
Eldreth et al. (2004)	H_2_^15^0 PET	11/11	1 (-)	Stroop Task	25 (-)	7.5 (-)	15.7 (-)	34.7/w	HPC	L ACC, L lateral PFC	No significant change
Kanayama et al. (2004)	fMRI	12/10	r6-36h	Spatial Working Memory Task	37.9 (7.4)	-	-	19,200 (-)/lifetime	PFC, ACC, basal ganglia	-	No significant change
Bolla et al. (2005)	H_2_^15^0 PET	11/11	28 (-)	Iowa Gambling Task	26 (-)	7.9 (-)	-	41 (-)/w	L cerebellum (Moderate Users>Heavy Users)	R OFC, R DLPFC (Moderate Users>Heavy Users)	↓Performance score
Gruber & Yurgelun-Todd (2005)	fMRI & DTI	9/9	-	Stroop Task	26.8 (3.6)	-	14.1 (-)	39.4 (-)/w	Midcingulate cortex	ACC	↑Commission errors
Chang, Yakupov, et al. (2006)	fMRI	24 [12 abstinent, 12 active]/19	r4-24h	Visual Attention Task	27.9 (10.8) [active], 29.6 (8.7) [abstinent]	-	15.5 (0.9) [active], 14.7 (0.4) [abstinent]	27.9 (1.1) [active], 26.7 (1.4) [abstinent]/m	Various frontal, parietal, occipital regions	R PFC, medial and dorsal parietal cortex, medial cerebellar regions (cerebellar changes normalised with abstinence)	No significant change
Jager et al. (2006)	fMRI	10/10	>7	Selective Attention Task	22.7 (4.2)	7.1 (3.9)	-	350 (-)/y [median]	No Significant Changes		No significant change
Hester et al. (2009)	fMRI	16/16	1.60 (2)	Go/No-Go Task	24.6 (1.5)	8.2 (1.3)	16.4 (0.7)	76.3 (17.7)/m	-	ACC, R insula	↓Error awareness
Abdullaev et al. (2010)	fMRI	14/14	2 (-)	Attention Network Task, Use Generation Task	19.5 (0.8)	5.1 (-)	14.7 (-)	132 (-)/y	R PFC	-	↑Reaction time, ↑ Errors
Becker et al. (2010a)	fMRI	26 [early-onset <16y cannabis users]/17 [late-onset >16y]	-	Verbal Working Memory	21.0 (2.8) [early onset], 24.5 (3.4) [late onset]	4.48 (3.4) [early onset], 3.88 (2.6) [late onset]	13.9 (1.0) [early onset], 17.0 (1.5) [late onset]	17.2 (10.7) [early onset], 9.8 (9.9) [late onset]/m	L superior parietal lobe (early-onset)	-	↑Reaction time in early-onset on 1-back task
Jager et al. (2010)	fMRI	21/24	35.7 (29.4)	Rule Based Learning	17.2 (1.0)	-	13.2 (2.3)	741.0 (772.0)/y	Prefrontal regions (novel task vs automised task)		No significant change
Vaidya et al. (2012)	H_2_^15^0 PET	46/38	1 (-)	Iowa Gambling Task	24.3 (3.9)	6.2 (3.2)	16.4 (1.9)	24.6 (6.2)/m	VMPFC, cerebellum	-	No significant change on standard IGT, ↓performance on variant IGT
Cousijn et al. (2013)	fMRI	32/41 Baseline, 6m	1.6 (2.2)	Iowa Gambling Task	21.9 (2.4)	2.9 (2.0)	-	4.9 (2.1)/w	R OFC, R insula, L superior temporal gyrus	-	No significant change
Filbey & Yezhuvath (2013)	fMRI	44 [D]/30 [ND]	3 (-)	Stop Signal Task	23.7 (6.5) [D], 24.8 (8.2) [ND]	5.5 (5.5) [D], 7.7 (7.5) [ND]	17.3 (2.5) [D], 17.4 (2.6) [ND]	3.4 (2.0) [D], 4 (4.0) [ND]/d	R frontal-control network, substantia nigra-subthalamic nucleus network	-	No significant change
Asmaro et al. (2014)	EEG & fMRI	13/15	1 (-)	Stroop Task	22.3 (3.0)	-	-	5.8 (1.6)/w	EEG: Early positive enhancement L frontal scalp, posterior/fMRI: L VMPFC, MOFC.	-	↓Accuracy (drug-containing blocks)
Behan et al. (2014)	fMRI	17/18	-	Go/No-Go Task	16.5 (0.2)	-	13.0 (0.2)	178.4 (38)/m	Parietal-Cerebellar Network	-	↓Accuracy
Cousijn et al. (2014)	fMRI	32/41 Baseline, 6m	1.8 (2.3)	N-back Working Memory Task	21.9 (2.4)	3.0 (1.9)	18.9 (2.4)	4.9 (2.1)/w	Working-Memory Network (VLPFC, DLPFC, premotor cortex, paracingulate cortex, inferior parietal cortex) - predicted weekly cannabis use at 6 months	-	No significant change
Colizzi et al. (2015)	fMRI	91/117 [CNR1 rs1406977 AA subjects/G carriers]	-	2-Back Working Memory Task	26.7 (6.3)	93.2% used for >5 years [AA subjects], 93.75% used for >5years [G carriers]	25.0 (42.4) [AA subjects], 10 (31.25) [G carriers]	-	L VLPFC (G allele carriers)	-	↓Accuracy (G carriers)
Gruber et al. (2017)	fMRI	45 [medical cannabis users]/0 Baseline, 3m	No abstinence	Multi-Source Inference Test (MSIT)	50.6 (13.2)	-	-	5.3 (2.0)/w	ACC	Normalisation of aberrant BOLD signal at 3 months vs baseline	↑Performance at 3m
Tervo-Clemmens et al. (2018)	fMRI	14 [occasional users]/46 [chronic users]/15 [non-users]	-	Working Memory Task	28.2 (0.7)	-	15.1 (2.3)	1.4 (2.7)/d	DLPFC	PCC (correlates with age of onset of cannabis use)	Overall ↑ performance in cannabis users, ↑Reaction times (earlier age of onset vs later age of onset)

Chronic effects on motor performance											
Pillay et al. (2004)	fMRI	9/16	r0.3-1.5	Finger Sequencing	37.3 (6.7)	21.0 (4.9)	18.4 (5.9)	-	-	SMA	-
Murphy et al. (2006)	fMRI	20/25	-	Finger Tapping Task	23.0 (-)	6.5 (-)	-	6 (-)/w	No Significant Changes		-
Pillay et al. (2008)	fMRI	11/16	28 (-)	Finger Tapping Task	37.7 (6.2)	-	-	-	-	SMA	-
King et al. (2011)	fMRI	30/30	0.5 (-)	Multiple Psychomotor/Motor Tasks	21 (-) [M], 22.5 (-) [F]	6.5 (-) [M], 5.3 (-) [F]	14.5 [M], 16.0 [F]	6.5 (-)/w	SMA	-	↓Psychomotor speed (M only)

ACC = anterior cingulate cortex, BOLD = blood oxygen level dependent, CNR1 = cannabinoid receptor 1 gene, d = day, D = dependent users, DLPFC = dorsolateral PFC, DTI = diffusion tensor imaging, EEG = electroencephalography, F = female, fMRI = functional magnetic resonance imaging, h = hour, HPC = hippocampus, IGT = Iowa Gambling Task, L = left, m = month, M =male, MOFC = medial orbitofrontal cortex, ND = non-dependent users, OFC = orbitofrontal cortex, PCC = posterior cingulate cortex, PET = positron emission tomography, PFC = prefrontal cortex, r = range, R = right, SMA = supplementary motor area, SD = standard deviation, VLPFC = ventrolateral prefrontal cortex, VMPFC = ventromedial prefrontal cortex, w = week, y = year.

### 4.7 Motor performance

Studies have used finger-sequencing and finger-tapping to measure fine motor function. Cannabis use was associated with impaired psychomotor performance and increased supplementary motor cortex activation in one study (King et al., 2011). However, when studying withdrawal from cannabis there is evidence (Pillay et al., 2004) of decreased task-induced activation in supplementary motor area which persists to 28 days of cessation (Pillay et al., 2008). However, these findings were not replicated in a separate study (Murphy et al., 2006) (Table 6).

### 4.8 Reward processing

Cross-sectional studies using the MID task have provided mixed results. There is evidence of ventral striatal hyperactivity during reward anticipation (Nestor et al., 2010) and putamen and caudate hyperactivity during anticipation of neutral trials (Jager et al., 2013). However, other studies have not found differences between cannabis users and controls on striatal response to reward anticipation (Enzi et al., 2015; Karoly et al., 2015) or report a blunted caudate response to reward anticipation in chronic cannabis users compared to non-smoking and smoking control groups (van Hell et al., 2010). Importantly, a longitudinal study following 108 volunteers at age 20, 22 and 24 years found that cannabis use was associated with blunted NAc response to reward anticipation at subsequent time points; there was no evidence for associations in the reverse direction (Martz et al., 2016).

In terms of feedback trials on the MID task, cross-sectional findings have also been mixed. Cannabis users have shown a blunted response to reward feedback in the left caudate and inferior frontal gyrus (Enzi et al., 2015) and increased right putamen response to reward feedback relative to smokers and non-using controls (van Hell et al., 2010). However, other studies have not found differences between cannabis users and controls in reward feedback, but instead have found striatal hyperactivation during reward anticipation (Jager et al., 2013). There is also evidence for blunted response to reward loss and loss avoidance in the left insula (Nestor et al., 2010). Blunted responses to reward loss may be clinically relevant, as ventral striatal hyperactivation during loss feedback predicted abstinence at 21 days in a group of dependent users following behavioural treatment for cannabis cessation (Yip et al., 2014). In an fMRI task of passive listening to preferred and neutral instrumental music (Ford et al., 2014) cannabis users did not show significant differences in activation compared to non-users and people experiencing depression. However, depressed cannabis users exhibited increased activation to preferred music in the putamen, anterior cingulate and right frontal regions compared to non-users and non-depressed users. This suggests that depression associated with cannabis use may be associated with disrupted reward processing (Table 7).

**Table 7:** Neuroimaging studies of the chronic effects of cannabis on reward processing, learning and memory, and emotional processing.

Author	Imaging Modality	Users/Controls (n) unless otherwise stated	Pre-trial abstinence, mean days (SD) unless otherwise stated	Activity	Mean User Age (SD)	Duration of use, mean years (SD)	Use onset age (SD)	Use frequency in joints/cones/uses, mean (SD) unless otherwise stated	Increase (volume, blood flow, activation, connectivity)	Decrease (volume, blood flow, activation, connectivity)	Task Performance (cannabis user vs comparison group)
Chronic effects on reward processing											
Nestor et al. (2010)	fMRI	14/14	9 (-)	Monetary Incentive Delay Task	23.1 (1.2)	6.1 (-)	16.1 (0.4)	7,258 (-)/lifetime	Ventral striatum	-	No significant change
Van Hell et al. (2010)	fMRI	14 [cannabis smokers]/14 [tobacco smokers]/13 [non-smoking controls]	>7	Monetary Incentive Delay Task	24.0 (4.4)	-	-	3841 (2645.3)/lifetime	R putamen (during reward feedback) (cannabis smokers vs tobacco smokers and non-smokers)	NAc (cannabis and tobacco smokers vs non-smokers), caudate (cannabis smokers vs tobacco smokers and non-smokers) (during reward anticipation)	No significant change
Jager et al. (2013)	fMRI	21/24	35.7 (29.4)	Monetary Incentive Delay Task	17.2 (1.0)	-	13.2 (2.3)	4,006 (7,555)/lifetime	Striatum (anticipation of neutral trials)	-	No significant change
Ford et al. (2014)	fMRI	15 [cannabis users]/15 [MDD]/14 [cannabis users with MDD]/17 [healthy controls]	-	Music Listening Paradigm (Neutral and Preferred Music)	20.2 (1.3) [cannabis users], 19.9 (1.7) [MDD + cannabis users]	6.8 (0.4) [cannabis users] 6.9 (0.4) [cannabis users + MDD]	-	22.0 (6.2) [cannabis users], 20.5 (9.2) [cannabis users + MDD]/m	Putamen, ACC, R frontal regions (preferred music, depressed cannabis users)	-	No significant change
Yip et al. (2014)	fMRI	20/20 [measured at 21 days of abstinence]	20 (-)	Monetary Incentive Delay Task	26.7 (2.2)	14.4 (3.3) [abstinent], 8.7 (1.9) [non-abstinent]	13.4 (0.5) [abstinent], 14.1 (0.6) [non-abstinent]	-	Ventral striatum (response to loss of reward, predicted abstinence at 21 days)	-	No significant change
Enzi et al. (2015)	fMRI	15/15	1.1 (1.1)	Monetary Incentive Delay Task	26.3 (2.9)	8.5 (3.0)	15.8 (2.7)	13.3 (7.3)/w	L caudate, inferior frontal gyrus	-	No significant change
Karoly et al. (2015)	fMRI	14 [cannabis users]/34 [tobacco only]/12 [alcohol only]/17 [cannabis + tobacco]/17 [cannabis + tobacco + alcohol]/38 [non-using controls]	>0.1	Monetary Incentive Delay Task	15.8 (1.4) [cannabis users], 15.8 (1.2) [cannabis + tobacco], 15.9 (1.0) [cannabis + tobacco + alcohol]	-	12.9 (1.9) [cannabis only], 11.4 (2.1) [cannabis + tobacco], 10.5 (2.6) [cannabis + tobacco + alcohol]	20.4 (8.9)/m [cannabis only], 24.4 (6.5)/m [cannabis + tobacco], 24.8 (6.9)/m [cannabis + tobacco + alcohol]	No Significant Changes (cannabis users vs other groups)		No significant change
Martz et al. (2016)	fMRI	108/0 (longitudinal cohort at age 20, 22, 24, cross-lagged model)	>2	Monetary Incentive Delay Task	20.1 (1.4), 22.1 (1.5), 23.8 (1.7)	-	15.4 (53.9) used cannabis by age 16	17.5 (58.1)/y [age 20], 30.4 (87.6)/y [age 22], 31.8 (89.9) [age 24]	-	NAc (reward anticipation)	No significant change

Chronic effects on learning and memory											
Block et al. (2002)	H_2_^15^0 PET	18/13	1.2 (0.0)	Word List Learning	-	-	-	18 (2)/w	Cerebellum/Altered lateralisation in HPC	PFC	↓Performance
Jager et al. (2007)	fMRI	20/20	-	Pictorial Memory Task	24.5 (5.2)	-	-	1,900 (-)/lifetime	-	Parahippocampal regions, R DLPFC	No significant change
Nestor et al. (2008)	fMRI	14/14	3.4 (2.0)	Face-Name Pairs Task	24.4 (1.4)	7.2 (1.1)	17.0 (0.9)	19.1 (2.7)/m	Parahippocampal gyrus	R superior temporal gyrus, R superior frontal gyrus, R middle frontal gyrus, L superior frontal gyrus	No significant change in fMRI experiment (n=14), but ↓performance in chronic users (n=35) in preliminary experiment
Becker et al. (2010b)	fMRI	42 [21 high frequency users, 21 low frequency users]/0	86.5 (235.7)	Face Encoding & Retrieval Task	22.5 (3.5)	-	15.1 (2.0)	14.2 (11.0)/m	L parahippocampal gyrus (encoding, high frequency>low frequency)	-	No significant change
Sneider et al. (2013)	fMRI	10/18	0.5 (-)	Morris Water Maze Task	20.3 (3.6)	4.0 (2.4)	15.6 (1.2)	10.7 (5.5)/w	-	R parahippocampal gyrus, cingulate gyrus	↓Memory Retrieval
Carey et al. (2015)	fMRI	15/15	4.2 (1.6)	Paired Associate Learning Task	22.4 (4.3)	6.4 (1.1)	16.0 (0.4)	72.5 (12.6)/m	-	dorsal ACC, L HPC	↓ Recall error-correction rate
Riba et al. (2015)	fMRI	16/16	≧28	Modified Deese-Roediger-McDermott paradigm	-	21 (-), r3-39	17 (-), r12-20	5 (-) [r1-24]/d	-	lateral and medial temporal lobe, parietal regions, frontal regions	↑Susceptibility to false memories

Chronic effects on emotional processing											
Gruber et al. (2009)	fMRI	15/15	>0.5	Viewing Happy/Fearful Faces	25.0 (8.8)	-	14.9 (2.5)	25.6 (27.8)/w	-	ACC, amygdala	-
Zimmermann et al. (2017)	fMRI	23/20	3.6 (1.8)	Cognitive Emotion Regulation Paradigm	21.24 (2.6)	4.3 (2.8)	16.0 (2.0)	5.7 (1.4)/w	frontal network (precentral, middle cingulate cortex, SMA), amygdala-DLPFC connectivity	-	↓Emotional regulation success
Zimmermann et al. (2018)	fMRI	21/20	167.0 (280.1)	Emotional Processing Paradigm	23.8 (3.2)	5.9 (2.9)	14.9 (1.3)	27.3 (5.9)/m	MOFC, MOFC-dorsal striatum, MOFC-amygdala connectivity	-	No significant change

ACC = anterior cingulate cortex, d = day, DLPFC = dorsolateral PFC, fMRI = functional magnetic resonance imaging, h = hour, HPC = hippocampus, L = left, m = month, MDD = major depressive disorder, MOFC = medial orbitofrontal cortex, NAc = nucleus accumbens, PET = positron emission tomography, PFC = prefrontal cortex, r = range, R = right, SMA = supplementary motor area, SD = standard deviation, w = week, y = year.

### 4.9 Learning and memory

Chronic cannabis use has been associated with negative effects across learning and memory including impaired recall (reviewed by Bossong et al. (2014) and Broyd et al. (2016)). Several mechanisms may be underlying this in addition to working memory dysfunction described earlier. For example, impaired error-related learning is associated with hypoactivity of the anterior cingulate and left hippocampus in cannabis users (Carey et al., 2015). A study using H_2_[^15^O]-PET found that chronic cannabis users have lower prefrontal blood flow and altered hippocampal lateralization during memory processing (Block et al., 2002). There is evidence that cannabis users and recently abstinent users exhibit parahippocampal dysfunction during encoding and retrieval (Becker et al., 2010b; Jager et al., 2007; Nestor et al., 2008). Episodic memory dysfunction in cannabis use, including increased risk of false memories, has been related to altered medial temporal lobe morphology (Smith, Cobia, et al., 2015) and function (Riba et al., 2015). In terms of spatial memory, compared to controls, cannabis users had right parahippocampal hypoactivation during a virtual water maze (Sneider et al., 2013) (Table 7).

### 4.10 Emotional processing

Cannabis users show behavioural impairments in the recognition of facial affect (Platt et al., 2010) and these were found to be robust after accounting for sex differences and schizotypal personality traits (Hindocha et al., 2014). Studies in adult heavy and regular cannabis users have found decreases in BOLD response within the cingulate, frontal cortex and the amygdala including during negative emotional stimuli presentation (Gruber et al., 2009; Zimmermann et al., 2017). This was alongside hypoconnectivity between the amygdala and DLPFC in active users and orbitofronto-striatal and amygdalar hyperconnectivity following 28 days of abstinence (Zimmermann et al., 2018) (Table 7).

### 4.11 CB_1_ receptor availability

Though the regional brain pattern of reduction in CB_1_R availability differed between studies, active cannabis use is associated with reduced CB_1_R availability that appears to normalise after abstinence. The first study (Hirvonen et al., 2012) measured CB_1_R binding using the selective radioligand [^18^F]FMPEP-d2 in 30 heavy cannabis users compared to 28 controls. This showed a 20% reduction in binding in the neocortex and limbic cortex of cannabis users which normalised after 4 weeks of monitored abstinence. The former finding was supported by a subsequent PET study (Ceccarini et al., 2015) of 10 chronic cannabis users using the CB_1_R inverse agonist radiotracer [^18^F]MK-9470 which showed a global 11.7% decrease in availability compared to controls. Region-of-interest analysis showed significant reductions in CB_1_R expression in the temporal lobe, ACC, PCC and NAc. A greater reduction in a similar study (D'Souza et al., 2016) using a different CB_1_R specific ligand ([^11^C]OMAR) demonstrated a 15% reduction in CB_1_R availability in limbic, cortical and striatal brain regions at 8-12 hours after last cannabis exposure. This reduction then rapidly normalised with non-significant reductions in CB_1_R availability evident after only two days abstinence.

### 4.12 The dopaminergic system

Several studies have imaged dopaminergic function in cannabis users. Using PET, striatal dopamine synthesis capacity was reduced in cannabis users and this was driven by users who were dependent on the drug (Bloomfield, Morgan, Egerton, et al., 2014). Importantly, within users, motivation levels were related to striatal dopamine synthesis capacity in the associative striatum (Bloomfield, Morgan, Kapur, et al., 2014). Two further studies using PET showed a reduction in striatal dopamine release in cannabis users in response to amphetamine challenge (van de Giessen et al., 2017; Volkow et al., 2014), however, a consistent pattern was not observed in recently abstinent cannabis users (Urban et al., 2012) suggesting this reduction is dependent on active use. The reduction in dopamine release also correlated with cognitive deficits including poor working memory (van de Giessen et al., 2017). These findings were supported by another PET study showing reduced metabolic response in the striatum in cannabis users after a methylphenidate challenge (Wiers et al., 2016). Another study that examined the interaction between chronic cannabis use and stress-induced dopamine release found no significant alteration in dopamine release, but did find a significant positive correlation between duration of cannabis use and dopamine release in the limbic striatum (Mizrahi et al., 2013). Further evidence of reduced dopaminergic activity in cannabis users came from PET imaging to examine dopamine transporter availability, showing lower dopamine transporter availability in the ventral striatum, the midbrain, the middle cingulate and the thalamus (ranging from -15 to -30%; Leroy et al., 2012). Several studies (Urban et al., 2012; van de Giessen et al., 2017; Volkow et al., 2014) have shown no significant striatal dopamine 2 receptor (D_2_R) availability differences between cannabis or ex-cannabis users and cannabis naïve participants. Nonetheless, one study (Albrecht et al., 2013) found a strong negative association between D_2_R availability and level of current cannabis use suggesting a potential dose-dependent effect. Similarly, another study (Urban et al., 2012) found a negative relationship between D_2_R availability and age of first use.

### 4.13 Glutamatergic and GABAergic systems

Five studies have investigated in vivo differences in glutamate-related metabolites in cannabis users (Colizzi et al., 2016). All of these studies used ^1^H magnetic resonance spectroscopy (MRS) in chronic cannabis users versus controls. The first study to do this (Chang, Cloak, et al., 2006) found a 9.5% reduction in basal ganglia glutamate metabolite levels in 24 daily cannabis users in comparison to 30 non-using controls. This study used the same model of analysis to look at frontal white matter glutamate metabolite levels in a sample including 42 people who were human immunodeficiency virus positive, half of whom were cannabis users, compared to 24 healthy cannabis users and 30 that were cannabis naïve (total n = 96). This further analysis showed even greater reductions (12-13%) in glutamate metabolite levels in chronic cannabis users, with healthy cannabis users having lower levels. The reduction in glutamate metabolite levels found in the basal ganglia and frontal white matter was also shown by two different studies (Prescot et al., 2011; Prescot et al., 2013) from the same research team (2011, n=34; 2013, n = 29) that found a similar 15% reduction in glutamate signal in the ACC and a concomitant reduction in GABA signal. However, these reductions in the same brain region were not found in another study (Sung et al., 2013), though this had a smaller sample size (n=8) and subjects were concurrently using methamphetamine. Only one imaging study to date (Muetzel et al., 2013) has looked at glutamate profiles of heavy cannabis users (n=27) versus healthy controls (n=26) in the striatum. This found no significant reduction in glutamate levels in the dorsal striatum but did find lower levels of glutamate and glutamine in female cannabis users but not males, compared to controls, suggesting a possible sex related difference.

These samples differed with respect to period of abstinence from cannabis prior to imaging. The first study (Chang, Cloak, et al., 2006) had no specific criteria regarding abstinence from cannabis use prior to scanning while another sample (Muetzel et al., 2013) only included those who were abstinent for over 12 hours. The two studies (Prescot et al., 2011; Prescot et al., 2013) showing significant reductions in glutamate metabolite levels in the ACC reported 54% of cannabis using participants had used cannabis in the preceding 24 hours. This could lead to significant variation in THC levels in the brain and animal studies have shown paradoxical outcomes on glutamate levels dependent on acute or chronic exposure to THC (Castaldo et al., 2010). Participants also differed significantly with regard to existing psychopathology. Three studies (Muetzel et al., 2013; Prescot et al., 2011; Prescot et al., 2013) included participants who had existing mental health problems, the first two of which included participants receiving antidepressant treatment for depression, which could impact glutamatergic systems (Duman, 2014; Sanacora et al., 2012). Outcome metabolite measures with MRS imaging also differed significantly. Two studies (Muetzel et al., 2013; Sung et al., 2013) measured both glutamate and glutamine metabolites, while all others only accounted for glutamate. Measurements also varied with regard to correction comparison of metabolite levels differing between correcting against water (Prescot et al., 2011; Prescot et al., 2013), cerebrospinal fluid (Chang, Cloak, et al., 2006), total creatinine (Muetzel et al., 2013) or phosphocreatinine and creatinine (Sung et al., 2013).

### 4.14 Other systems

Using [^18^F]2-F-A-85830 PET, Broyd et al. (2016) found that tobacco smokers with concurrent heavy cannabis use (defined as over 22 days per months) had higher α4β2 nicotinic acetylcholine receptor availability than smokers without drug use. Interestingly, findings in cannabis using smokers were similar to those seen in heavy caffeine users. Given the very different pharmacology of cannabis and caffeine, this suggests that the increased nicotinic acetylcholine receptor availability in tobacco users may not be specifically mediated by heavy cannabis use.

Given the putative neurotoxic effects of cannabis (Pope et al., 2010), there is interest in the impact of heavy cannabis use on regional levels of N-acetylaspartate (NAA), a proxy marker of neuronal integrity (Moffett et al., 2007). The first MRS study on this subject found that the NAA to total creatine ratio was decreased in the DLPFC of heavy cannabis users versus controls (Hermann et al., 2007). A decrease in NAA to total creatine ratio was since replicated in the neighbouring inferior frontal gyrus of polydrug users, which was negatively correlated with degree of cannabis use only (Cowan et al., 2009), and the mid-frontal anterior cingulate area of methamphetamine and cannabis users versus methamphetamine users alone (Sung et al., 2013). These results suggest that heavy cannabis use may cause disruption of neuronal architecture in frontal structures. This corroborates findings of decreased orbitofrontal gyrus (Filbey et al., 2014) and ACC (Hill et al., 2016) volumes, decreased resting state CBF to the ACC (Wiers et al., 2016) and orbitofrontal gyrus (Sevy et al., 2008), and alterations in ACC (Carey et al., 2015; Ford et al., 2014), inferior frontal gyrus (Enzi et al., 2015) and DLPFC (Jager et al., 2007) activity during emotional processing, reward and learning in chronic cannabis users. Decreases in NAA were also reported in the hippocampus of cannabis users relative to controls, alongside a reduction in hippocampal volume (Yücel et al., 2016). However these findings were not present in those with evidence of CBD exposure, or in abstinent users. These findings are consistent with a protective role of CBD on hippocampal dependent memory (Englund et al., 2013; Morgan et al., 2010) and for recovery of impaired performance following abstinence (Schreiner & Dunn, 2012; Scott et al., 2018).

## 5 Developmental effects of cannabis

Key periods for brain development occur in utero and during adolescence. Importantly, prenatal exposure to cannabis may produce persistent effects on working memory and executive function in adulthood (Smith et al., 2006; Smith et al., 2016). Given the potential of multiple confounds associated with investigating the effects of in utero drug exposure and effects which are very distal to the exposure, further larger prospective studies are needed to corroborate these findings given the potential public health impact of consuming cannabis during pregnancy and breast-feeding.

Heavy cannabis use during adolescence likely represents a critical period of vulnerability to cannabis-induced changes in brain function because the brain undergoes significant developmental changes at this age (Choudhury et al., 2006). Hippocampal hypertrophy has been associated with adolescent cannabis use (mean age 17 years, mean exposure duration two years) (Medina et al., 2007), although this was not found by Gilman, et al. (2014). Findings of increased grey matter density in other limbic subcortical structures in young cannabis users may reflect cannabis-induced changes in arborisation (Gilman et al., 2014). In parallel, there is some evidence of a relationship between prefrontal volume and executive dysfunction in adolescent users (Medina et al., 2009). These structural findings were extended by a study (Ashtari et al., 2009) of young male heavy cannabis users who, compared to non-users, had reduced frontotemporal structural connectivity via the arcuate fasciculus. Importantly, there is longitudinal evidence of structural hypoconnectivity associated with cannabis use in adolescents (Epstein & Kumra, 2015). In terms of functional connectivity, a large study (Thijssen et al., 2017) in adolescents found a relationship between duration of cannabis use and reduced functional connectivity within the default mode, executive control and auditory networks. In a study of adolescents admitted for treatment of cannabis dependence, the level of dependence was associated with reduced interhemispheric yet increased right intrahemispheric resting functional connectivity (Orr et al., 2013). Some studies have investigated the functional significance of dysconnectivity. For example, in young male long-term heavy cannabis users, drug use was associated with reduced striato-frontal connectivity (Blanco-Hinojo et al., 2017). These connectivity alterations were associated with lower arousal in response to affective pictures as measured with the International Affective Picture System and normalized after abstinence. A separate, longitudinal study of resting functional connectivity in adolescents demonstrated dysconnectivity between the caudal ACC, dorsolateral and orbitofrontal cortices over an 18 month follow-up period (Camchong et al., 2017). Amounts of cannabis use during this period were associated with inattention and impaired cognition. Another study found greater bilateral amygdalar activity during emotional processing, rather than the reduction seen in adults, to angry faces rather than neutral faces in 70 adolescent cannabis users (Spechler et al., 2015). However, this may simply be because the adolescent participants in Spechler’s sample had very minimal exposure in comparison to studies of heavy adult users. These studies suggest that adolescence may be a particularly critical time for cannabis’ effects on emotional and cognitive function. These findings are in keeping with a recent literature review suggesting that early, heavy cannabis use in adolescence predicts poor emotional processing and cognition in adulthood (Levine et al., 2017).

However, the significance of these neuroimaging findings relative to cognitive performance is unclear. A systematic review in 2016 found that whilst adolescent heavy cannabis users have radiological evidence of dysconnectivity, their performance in cognitive tasks is similar to controls (Lorenzetti, Alonso-Lana, et al., 2016). This led the authors to question whether functional dysconnectivity in these adolescents is caused by cannabis use, or is an adaptation that affords normal cognitive functioning. Further longitudinal studies are needed to clarify the significance of cannabis use in adolescence on cognition (James et al., 2013). Moreover, experimental, placebo-controlled studies are warranted. The only study to date (Mokrysz et al., 2016) found that adolescent cannabis users showed a profile characterised by resilience to some acute effects of cannabis (memory impairment, psychotic-like symptoms) and vulnerability to others (lack of satiety, impaired inhibitory processing).

Cognitive task performance may alter with abstinence (Scott et al., 2018). Abstinent adolescent cannabis users showed left orbitofrontal hypoactivation to non-reward vs. risky rewards which was related to cannabis use duration (De Bellis et al., 2013) whereas a separate study found evidence of fronto-parietal hyperactivation during response inhibition (Tapert et al., 2007). Whilst causal inferences are limited, these findings would be in keeping with increased incentive salience toward riskier rewards alongside less efficient response inhibition – which may be related to addictions generally and not specifically cannabis use.

There is consistent preclinical and neuropsychological evidence for cognitive effects of cannabis use during adolescence (Jager & Ramsey, 2008; Schweinsburg et al., 2008). Adolescents exhibit a similar pattern to adults of task performance and brain activity associated with non-acute cannabis effects (Bossong et al., 2014). Adolescent cannabis use is associated with increases in brain activity in prefrontal and parietal brain areas (Jacobsen et al., 2007; Jager et al., 2010; Schweinsburg et al., 2008; Schweinsburg et al., 2010) which may reflect reduced cortical efficiency. Adolescent cannabis use is also associated with greater task-induced de-activation (Schweinsburg et al., 2008; Schweinsburg et al., 2005; Schweinsburg et al., 2010) which is consistent with increased effort to maintain task performance. Comparisons between adult and adolescent studies are limited by lower cumulative exposure, lower duration of exposure in adolescents than in adults alongside differences in durations of abstinence. Nonetheless, it remains possible that the effects of cannabis use on the adolescent brain may be more harmful given the potential to alter developmental trajectories (Bossong & Niesink, 2010; Curran et al., 2016).

## 6 Cannabis use disorders

Based on population-based data from the United States in 2012-2013, the past year prevalence of cannabis use disorders was estimated at 2.9%, or 30.6% among past-year users (Hasin et al., 2015). Given the high rate of cannabis use worldwide, estimated at 183 million past year users (United Nations Office on Drugs and Crime (UNODC), 2018), a substantial number of people currently meet criteria or at risk of developing a cannabis use disorder. In terms of clinical implications, cannabis now accounts for around half of all first-time entrants to specialist drug treatment worldwide (United Nations Office on Drugs and Crime (UNODC), 2018) and has now superseded opiates as the primary reason for first-time treatment entry of all illicit drugs in Europe (European Monitoring Centre for Drugs and Drug Addiction (EMCDDA)., 2018). One possible contributor to the increase in cannabis-related treatment admissions may be the increase potency of cannabis products, resulting in a higher dose of THC and greater harm to users. A 16-year study in the Netherlands found that changes in the THC concentration of cannabis sold in national retail outlets were positively associated with the number of people subsequently entering treatment for cannabis problems (Freeman, van der Pol, et al., 2018). Psychological interventions such as Cognitive Behavioural Therapy and Motivational Interviewing have limited effectiveness, and there are no approved pharmacotherapies available.

The high density of CB_1_Rs in reward and habit circuits, and the key role of the endocannabinoid system in reinforcement may underpin the effects of THC in the development, withdrawal and relapse of cannabis use disorders (Curran, et al., 2016). Chronic THC exposure is associated with downregulation of CB_1_Rs (Ceccarini et al., 2015; D'Souza et al., 2016; Hirvonen et al., 2012). Moreover, withdrawal from chronic cannabis administration is associated with reduced dopamine transmission in the NAc (Diana et al., 1998) and the reduction in striatal dopamine synthesis capacity shown found in cannabis users was driven by those meeting clinical Diagnostic & Statistic Manual of Mental Disorders IV criteria for cannabis use disorders (Bloomfield, Morgan, Egerton, et al., 2014). Evidence for blunting of the dopamine system in cannabis use disorders (Bloomfield et al., 2016) is consistent with prospective evidence from a longitudinal analysis of adults aged 20, 22, and 24 (Martz et al., 2016). That study found that cannabis use predicted a blunted NAc response to reward anticipation at subsequent time points. If cannabis use dampens anticipatory reward processing over time, as suggested by this study, chronic use may increase vulnerability to mental health disorders across diagnostic categories including addiction to other substances and gambling (Luijten et al., 2017) depression and psychosis (Hagele et al., 2015).

## 7 Cannabis and psychoss

 When considering the links between cannabis use and psychosis it is important to remember that the schizophreniform clinical syndrome lies at a confluence of phenotypes including hallucinations, paranoia, amotivation and cognitive impairment. All of these have been associated with acute exposure to THC (Bhattacharyya et al., 2010; Broyd et al., 2016; Curran et al., 2016; D'Souza et al., 2004; Moreau, 1845; Morrison & Stone, 2011; Morrison et al., 2009) and long-term heavy cannabis use (Broyd et al., 2016; Curran et al., 2016; Freeman et al., 2013; Marconi et al., 2016) in vulnerable individuals. Cannabis produces complex neuropharmacological effects on systems underlying these experiences. There are several important findings that stand out which relate to executive function, memory and the limbic system. For example, THC alters the neural response during working memory performance (Böcker et al., 2010; Bossong, Jager, et al., 2012) as seen in schizophrenia (Sutcliffe et al., 2016). Likewise, psychosis is associated with altered threat processing (Freeman et al., 2013) and THC produces complex effects on neural systems underlying fear processing including altered amygdalar response to threat and reduced amygdalo-cortical coupling (Gorka et al., 2015), and THC may be anxiogenic via non-amygdalar pathways. Recent work has shown that CB_1_Rs are involved in midbrain threat processing (Back & Carobrez, 2018) and further work is needed to understand the potential involvement of these pathways in the pathophysiology of psychosis. Structurally, changes associated with early onset heavy use include hippocampal (Rocchetti et al., 2013) and amygdalar atrophy (Lorenzetti et al., 2015) alongside aberrant self-processing and executive network connectivity (Cheng et al., 2014; Filbey & Dunlop, 2014; Lopez-Larson et al., 2015; Orr et al., 2016), which map conceptually onto schizophreniform symptomatology. At the molecular level, heavy cannabis use is associated with perturbations of the endocannabinoid system (D'Souza et al., 2016). The development of clinical schizophrenia following heavy use may be through non-hyperdopaminergic processes (Bloomfield et al., 2016) in contrast to idiopathic schizophrenia (Howes & Kapur, 2014), with potential candidate mechanisms including excitatory-inhibitory imbalance between GABA-ergic (Radhakrishnan et al., 2015) and glutamatergic (Prescot et al., 2013) systems, which are intimately modulated by the endocannabinoid system. Together these neurocognitive, neurochemical and structural changes could therefore give rise to clinical schizophrenia in people who are vulnerable to the deleterious effects of cannabis use across the dimensions of the clinical syndrome.

Broadly speaking there are two possible explanations for this which are not mutually exclusive: (1) cannabis is exacerbating the same vulnerabilities that cause idiopathic schizophrenia and (2) cannabis causes additional routes to the phenotype. One of the first neuroimaging studies in cannabis and psychosis used CT (Wiesbeck & Taeschner, 1991) to compare a drug-using group of patients with psychotic symptoms to a non-using group of patients found no differences between the two groups. Subsequently, Cunha et al. (2013) found that cannabis using patients with first episode psychosis did not have grey matter volume deficits in the medial temporal lobe or PFC that were typical of psychotic patients without cannabis use suggesting that cannabis use induced psychosis via different neurodevelopmental pathways to idiopathic schizophrenia. In support of this, a small study (Dragogna et al., 2014) found that patients with cannabis-induced psychosis had hypermetabolism in the posterior cingulate and precuneus compared to patients with schizophrenia without cannabis use. In a study comparing white matter connectivity in adolescent-onset schizophrenia with and without cannabis use (over three times per week for at least six months) there was decreased fractional anisotropy in the internal capsule, corona radiata, superior and inferior longitudinal fasciculus (James et al., 2011). However, a previous study limited by small sample size (Peters et al., 2009) found contrary evidence. THC-induced effects have been extended to functional connectivity in patients with schizophrenia and co-morbid cannabis use disorder, assessed after seven days of abstinence (Fischer et al., 2014). At baseline, patients in this study had hypoconnectivity between the NAc and frontal reward regions including the OFC and ACC, which was reversed upon THC challenge. One possible explanation is that patients with schizophrenia may be motivated to use cannabis in order to restore their dysregulated brain reward circuitry. In addition, in a study of adolescents with early onset schizophrenia (Epstein et al., 2014), cannabis use was associated with impaired attention network function compared to patients without cannabis use disorder. Atakan et al. (2013) compared brain function between subjects who did (N=11) and did not (N=10) experience psychotic effects following oral THC administration (10 mg). THC showed stronger effects on inhibition errors in the group of participants with psychotic symptoms, accompanied by increased psychosis-related activity in the right middle temporal gyrus and decreased activity in the parahippocampal and fusiform gyri. Following this, a large study of patients at clinical high risk of schizophrenia (Buchy et al., 2015) examined the relationship between thalamic dysconnectivity and cannabis use. Whilst there was no discernible effects on thalamic connectivity based on current cannabis use status, there was some evidence that within patients at high clinical risk of schizophrenia who were also cannabis users, there was a relationship between thalamo-sensorimotor hypoconnectivity and age of onset of cannabis use.

Findings of differences between patients with psychosis with and without cannabis use (Cunha et al., 2013; Dragogna et al., 2014; James et al., 2011) may support the presence of a potentially distinct ecophenotypic subtype of schizophrenia secondary to heavy cannabis use which could have implications for prevention and treatment thereby necessitating further work to investigate how these differences relate to phenomenology on the one hand. On the other hand, understanding shared mechanisms has the potential to yield new treatment targets - which would be most welcome for a disorder which has seen minimal progress in meaningful new treatments since Kane’s pioneering work on clozapine 30 years ago (Kane et al., 1988).

## 8 Discussion

The large body of work reviewed indicates that cannabis can alter brain structure, interfere with executive function, subvert the reward system, and produce complex effects on emotional processing. A wide range of neuropharmacological systems likely underlie these effects including the endocannabinoid, dopamine, glutamate and GABA systems. The mounting evidence is testament to the importance and broad interest in the topic over the last few decades. The imaging methods used (from early volumetric CT studies, to contemporary functional imaging) are diverse, and many of the methods themselves have been undergoing significant development in the same time period. Beyond the experimental methods, the literature is extremely varied in a number of other factors including the participant population studied, route of administration and dose used (for acute challenge studies), and the definitions of usage (for studies of chronic users). All these factors present challenges to the construction of a coherent synthesis. Nonetheless, we have presented a number of themes and a set of relatively consistent results that we have seen emerge. We will now describe some of the methodological considerations that limit the interpretations that we have made from this field of research.

### 8.1 Pharmacological considerations

There are a range of factors that may account for disparities in the results between studies. Firstly, in some experiments participants were given cannabis, whereas in other studies pure THC was administered. Although THC is the main psychoactive ingredient, cannabis contains at least 144 phytocannabinoids (Hanuš et al., 2016), and therefore the acute effects of THC and cannabis are likely to be different. Secondly, studies applied different methods of administration with varying doses of THC, resulting in different pharmacokinetic and pharmacodynamic effects (Grotenhermen, 2003). Thirdly, oral consumption generally leads to slower absorption and lower bioavailability of THC, and a delay in the onset of acute behavioural effects compared to inhalation (Agurell et al., 1986; Grotenhermen, 2003). Finally, variation in the participants’ history of cannabis use between studies may have affected the findings, as frequent cannabis use may result in blunted responses to acute effects of cannabis (Curran et al., 2018; D'Souza et al., 2008). For studies on the chronic effects of cannabis, interpretation of the results is significantly hampered by large differences in characteristics of study populations. These include frequency, quantity, history and age of onset of cannabis use, time that subjects were abstinent from using cannabis, and rates of tobacco smoking, alcohol consumption and use of other illicit drugs. For the studies on the chronic effects of cannabis, differences in the composition of cannabis may also be important. The effects of cannabis appear to depend on the ratio between THC and CBD as both substances may have opposite neural effects during fMRI (Bhattacharyya et al., 2012; Bhattacharyya et al., 2010). Therefore, the composition of cannabis may have been a confounding factor when investigating non-acute effects of cannabis. The composition of cannabis has also changed over time (ElSohly et al., 2016; Pijlman et al., 2005; Potter et al., 2018; Zamengo et al., 2015) which may have affected the comparison of findings between studies as well as individual results within studies. Furthermore, definitions of what constitutes a “cannabis user” are highly inconsistent across studies and alongside this, consensus is needed in the field as to how to measure the amount of cannabis/THC being consumed i.e. an internationally agreed standard unit of THC and THC:CBD ratio for users, clinicians and scientists (Hindocha, Norberg, and Tomko, 2018). Lastly, there is the perennial challenge of retrospective recall of the amount of cannabis that is being consumed which can only be addressed through robust prospective designs.

### 8.2 Imaging considerations

The imaging methods used are diverse and range from early studies looking at volumetric measures with CT images, PET studies with various ligands, diffusion MRI, functional MRI, and even some EEG studies. Each of these methods has their own set of advantages and drawbacks that are generally relatively well-known and adequately described elsewhere. We will, therefore, focus on specific idiosyncrasies that apply to the literature reviewed above.

There is an emerging awareness that many neuroscience studies may be severely under-powered in a statistical sense (Button et al., 2013; Nord et al., 2017) and neuroimaging studies may be particular examples, because their relatively high cost (in both money, and researcher time) make collecting large samples difficult. Under-powered studies can produce false positive results (the “winner’s curse” effect; Button et al., 2013) that subsequently fail to replicate (Cremers et al., 2017) and over time this potentially leads to a large number of inconsistent results, and low reproducibility in the literature as a whole. Low power may be a particular issue in pharmacological neuroimaging research as many studies use between-subjects designs (e.g. comparing cannabis users and non-users), or within-subjects designs where the relevant comparisons are on different days and/or scan sessions (e.g. comparing placebo and active cannabis), sometimes weeks apart. Both of these designs inherently have higher noise levels (and therefore lower power) than a more ‘standard’ neuroimaging experimental design where, for example, active task and rest conditions are compared within a single scan session. In addition, neuroimaging is a rapidly evolving field, with major advancements continuing to be made in both acquisition (hardware and software) and analysis methods. These innovations mean that the acquisition and analysis procedures in methods such as fMRI are not fully standardised, and may not be for the foreseeable future. For example, in early fMRI studies it was relatively common to use uncorrected thresholds of p < 0.001 in group-level analyses (e.g. Kanayama, et al., 2004) but this would be deemed unacceptably lax in most modern studies. Recent high-profile work has highlighted somewhat more subtle, but important, statistical issues (Eklund et al., 2016) which may also contribute to the production of false-positive results in the literature. There is little practical utility in an exercise of formally re-assessing large sections of the literature in light of these advancements, however the enlightened reader should certainly bear these issues in mind when evaluating previous work, particularly the older studies, with relatively small numbers of subjects.

The methods continue to advance, and recent innovations such as lightweight, wireless EEG systems (Ratti et al., 2017), high field-strength MRI scanners (Duyn, 2012) highly accelerated scanning sequences for fMRI (Demetriou et al., 2018), machine-learning based analysis methods (Doyle et al., 2015) and combined PET/MR scanners (Sauter et al., 2010) are of great interest, but will also necessarily entail their own sets of caveats and compromises. Larger-scale publically-available data sets with many hundreds of subjects such as the Human Connectome Project (HCP; e.g. Pagliaccio, et al., 2015) and the UK Biobank (Sudlow et al., 2015) are also beginning to address the issues of small sample sizes and low experimental power. True standardisation of methods in human neuroimaging is unlikely while the field is undergoing such rapid and continuous advancement, but attempts to unite around common standards for at least some aspects of the procedures are making some headway (e.g. Esteban et al., 2018). All these developments are highly positive, and can only lead to higher-quality, more robust, and more reproducible future work.

### 8.3 The future

Great progress has been made in our understanding of the effects of cannabis and THC on the human brain. This progress will likely intensify, given the public health implications of heavy use, changes to the legal landscape of the drug and new medicines in the pipeline that will target the endocannabinoid system. Given the changing patterns of use, with heavy use appearing to carry the most risk, there is an urgent need to fully elucidate the effects of heavy cannabis use during development and their reversibility. Beyond THC, we must understand the diverse effects of the myriad of phytocannabinoids in cannabis and the synthetic cannabinoids that are being increasingly used recreationally. Likewise, we must reach a precise understanding of the neurobiological mechanisms underlying cannabis dependence and psychosis. This should include systematic multimodal imaging that can better update our understanding of such complex mechanisms than single neuroimaging methods. In parallel, greater understanding of these systems may offer hope to the many millions of people suffering from mental illnesses throughout the world in the form of new treatments.

## 9 Conclusions

There is a mounting body of evidence informing us of both the mechanisms underlying the psychoactive effects of THC and the long-term effects of cannabis use. The available evidence suggests the drug disrupts emotional processes, executive function and reward function via the endocannabinoid system which likely underlie the mental health problems associated with heavy cannabis use. While also informing the underlying pathophysiology of a range of disorders, improved understanding of these systems may lead to new treatment targets in the future. Both longitudinal studies and well-designed pharmacological challenges are needed to elucidate the precise effects of THC, CBD and the other major cannabinoids on the brain.

## Conflict of interest statement

Dr Wall’s primary employer is Invicro, a private company which performs contract research for the pharmaceutical and biotechnology industries. Otherwise, the authors declare that there are no conflicts of interest.
